# Single‐Cell Reveal GALNT7‐Dependent Ferroptosis Suppression as a Mechanism of Immunotherapy Resistance in Non‐Small Cell Lung Cancer

**DOI:** 10.1002/advs.76082

**Published:** 2026-06-19

**Authors:** Jiadi Gan, Qian Zheng, Deng Liu, Ruichao Nie, Menglin Yao, Xiaolong Tang, Renjie Xu, Jinghong Xian, Wenjun Meng, Wenxin Luo, Dan Liu, Guiyi Ji

**Affiliations:** ^1^ Department of Pulmonary and Critical Care Medicine Frontiers Science Center for Disease‐Related Molecular Network State Key Laboratory of Respiratory Health and Multimorbidity West China Hospital West China School of Medicine Sichuan University Chengdu Sichuan China; ^2^ Department of Anesthesiology The First Affiliated Hospital of Anhui Medical University Hefei Anhui China; ^3^ National Institute for Data Science in Health and Medicine Xiamen University Xiamen Fujian China; ^4^ Institute of Respiratory Health Frontiers Science Center for Disease‐related Molecular Network West China Hospital Sichuan University Chengdu Sichuan China; ^5^ Department of Pain Management West China Hospital Sichuan University Chengdu Sichuan China; ^6^ Health Management Center General Practice Medical Center West China Hospital Sichuan University Chengdu Sichuan China

**Keywords:** cancer research, cd8, gene knockdown, gene silencing, immune checkpoint, immunogenic cell death, immunotherapy, interferon gamma, t cell, tumor microenvironment

## Abstract

Immunotherapy has transformed the treatment of non‐small cell lung cancer (NSCLC), yet most patients fail to respond due to poorly understood resistance mechanisms. Here, integrative multi‐omics analysis combining single‐cell RNA‐sequencing, bulk RNA‐seq, and spatial transcriptomics is performed on tumors from NSCLC patients receiving immune checkpoint blockade (ICB). Transcriptomic profiling revealed that GALNT7, a glycosyltransferase, is selectively upregulated in non‐responders (NR) and enriched in malignant epithelial cells. Functional and pathway analyses linked GALNT7 expression to suppression of ferroptosis‐related signaling, whereas ICB responders (R) exhibited higher ferroptosis activity. Silencing GALNT7 in NSCLC cells impaired proliferation, induced apoptosis, and triggered ferroptotic cell death, characterized by lipid peroxidation and mitochondrial damage. Mechanistically, GALNT7 loss decreased SLC7A11 and GPX4, while upregulating the ferroptosis activator ACSL4. In vivo, GALNT7 knockdown reduced tumor growth, enhanced CD8^+^ T cell infiltration, and increased interferon gamma (IFN‐γ) production within the tumor microenvironment. Moreover, combining GALNT7 depletion with PD‐1 blockade achieved synergistic tumor suppression, which is reversed by Ferrostatin‐1, indicating ferroptosis‐dependent immunostimulation. Collectively, these findings uncover GALNT7 as a critical regulator linking ferroptosis and anti‐tumor immunity, providing a mechanistic basis for ferroptosis‐based sensitization to ICB therapy in NSCLC.

## Graphical abstract

Integrated clinical and mechanistic analyses identify GALNT7 as a ferroptosis‐suppressive regulator associated with immunotherapy resistance in non‐small cell lung cancer. GALNT7 depletion promotes lipid peroxidation, mitochondrial dysfunction, and ferroptosis, enhances CD8^+^ T‐cell activation and IFN‐γ production, and sensitizes tumors to PD‐1 blockade, revealing a potential strategy to overcome resistance.

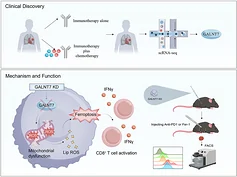

## 1 Introduction

Lung cancer continues to be the foremost cause of cancer‐related mortality worldwide, responsible for roughly 1.8 million deaths each year [1, 2]. Although therapeutic options for NSCLC have expanded over the past decade, the prognosis for most patients remains unsatisfactory. The introduction of ICB targeting PD‐1/PD‐L1 has transformed clinical practice, yet durable benefit is limited to a minority of cases [3, 4, 5]. Both primary non‐response and secondary relapse are now recognized as major clinical hurdles [6, 7]. Mechanistic studies suggest that antigen‐presentation defects [8], oncogenic signaling, metabolic rewiring, and an immunosuppressive tumor microenvironment (TME) each contribute to immune escape and resistance to ICB [8, 9]. Despite this, the fundamental molecular determinants that dictate immune escape are still incompletely defined. Ferroptosis, an iron‐dependent, lipid‐peroxidation–driven cell death pathway, has drawn attention for its potential to intersect with both tumor metabolism and immunity [10, 11, 12]. This process is controlled by regulators such as ACSL4, GPX4, and the cystine/glutamate antiporter system Xc‐ (SLC7A11/SLC3A2) [13]. Loss of ferroptosis competence has been implicated in tumor progression and therapy resistance across multiple malignancies [14, 15]. Using small molecules such as erastin (ferroptosis inducer), RSL3, or sorafenib to activate ferroptosis can selectively eliminate cancer cells that rely on aberrant redox balance [16].

Recent work has further illuminated bidirectional crosstalk between ferroptosis and the immune landscape. Activated CD8^+^ T cells, for instance, can induce ferroptosis in tumor cells through IFN‐γ–mediated repression of SLC7A11 [17], while ferroptotic tumor cells release oxidized lipids and DAMPs that may promote dendritic cell maturation and antigen presentation [10, 18, 19]. Conversely, excessive lipid peroxidation can also compromise T cell fitness, suggesting a finely tuned equilibrium between cytotoxicity and oxidative stress [20]. In several tumor models, ferroptosis enhances PD‐1 blockade efficacy and reshapes the tumor immune microenvironment (TIME) toward a more inflamed phenotype [21, 22]. However, how ferroptosis is regulated in NSCLC, and how this regulation intersects with ICB response remains unclear.

In view of these gaps, we combined single‐cell, bulk, and spatial transcriptomic analyses from NSCLC cohorts treated with immunotherapy to identify ferroptosis‐related determinants of resistance. Through this approach, we identified GALNT7, a glycosyltransferase involved in O‐glycosylation, as a ferroptosis suppressor enriched in NR. Functional studies demonstrated that GALNT7 depletion promotes lipid peroxidation, activates ferroptotic pathways, and enhances CD8^+^ T cell infiltration and IFN‐γ secretion, ultimately sensitizing tumors to PD‐1 blockade. These results delineate a previously uncharacterized GALNT7–ferroptosis–immunity axis, suggesting a potential therapeutic target for overcoming immunotherapy resistance in NSCLC.

## 2 Materials and Methods

### 2.1 Specimen Acquisition and Preparation

Following institutional review board–approved protocols and in accordance with CPTAC biospecimen standard operating procedures (SOPs) (see CPTAC SOP Compendium and corresponding prospective collection SOPs, https://brd.nci.nih.gov/brd/sop‐compendium/show/41?utm_source), All seven in‐house tumor tissues were treatment‐naïve at the time of collection before initiation of immunotherapy. Seven advanced NSCLC patients (first‐line ICI or adjuvant/post‐operative ICI) underwent biopsy or surgical resection prior to ICI initiation (Table S1A). No patients had received prior chemotherapy, radiotherapy, or immunotherapy before the sampled procedure. Resection specimens were transferred to liquid nitrogen within ≤ 30 min after removal. Hematoxylin and eosin (H&E) staining of the central block of each specimen was reviewed independently by two board‐certified pathologists to confirm diagnosis and tumor cellularity.

For cross‐cohort validation, 14 publicly available neoadjuvant ICI scRNA‐seq datasets were integrated (Table S1B). Clinical response labels were based on the definitions used in the original studies. In neoadjuvant settings, patients were classified as responders or non‐responders based on pathological complete response (pCR) or major pathological response (MPR) criteria, with residual viable tumor ≤ 10% classified as responders. In advanced disease cohorts, radiographic RECIST v1.1 criteria were applied, with complete response (CR) or partial response (PR) classified as R and stable disease (SD) or progressive disease (PD) as NR (Table S1A,B).

For the integrated human scRNA‐seq discovery cohort, we initially assembled institutional and publicly available ICI‐treated NSCLC datasets. To reduce cohort imbalance and ensure consistent response classification, we refined the cohort before downstream analysis. Specifically, the only institutional stage IVA scRNA‐seq case was excluded from the integrated cohort to minimize stage‐related heterogeneity. In addition, one public case was excluded after re‐review because its treatment‐response evaluation could not be assigned with sufficient confidence according to our predefined response‐classification criteria. All downstream single‐cell analyses were repeated using the refined cohort.

The final integrated human scRNA‐seq atlas therefore consisted of 21 tumors, including 7 in‐house scRNA‐seq cases and 14 publicly available ICI‐treated scRNA‐seq cases (Table S1A,B). One additional independent institutional non‐responder case was analyzed separately by spatial transcriptomics as an illustrative resistance‐focused spatial case study and was not included in the integrated scRNA‐seq cohort or in cross‐cohort statistical analyses (Table S1C).

### 2.2 Single‐Cell Dissociation, Library Construction and Sequencing

Fresh tumor tissues were minced and enzymatically dissociated using Collagenase IV (1 mg mL^−1^) and DNase I (50 U mL^−1^) in HBSS for 20–30 min at 37°C with gentle agitation, consistent with previously reported clinical lung cancer single‐cell workflows [23]. The tissue was then filtered through a 40‐µm strainer and subjected to red blood cell (RBC) lysis. Viable cells were quantified by Trypan blue exclusion, with a threshold of > 85% viability. To minimize ex vivo transcriptional perturbation, all in‐house samples were processed using the same dissociation workflow and within the same predefined time window. For the in‐house cohort, single‐cell RNA libraries were prepared using the 10x Genomics Chromium Single Cell 3′ v3.1 platform following the manufacturer's instructions. Sequencing was performed on the Illumina NovaSeq 6000 platform, targeting 50 000–80 000 reads per cell (paired‐end, 28 × 91 bp). Raw sequencing data were processed using Cell Ranger v6.1.2 with the GRCh38 reference genome.

### 2.3 Single‐Cell RNA‐seq Processing and Integration

All analyses were performed in R 4.2.3 using Seurat v4.3.0. Cells were retained if they met all QC thresholds: 500–6000 detected genes, 1000–40 000 UMIs, and < 15% mitochondrial RNA. This range is consistent with thresholds used in recent large‐scale human tumor single‐cell atlas studies [24]. The mitochondrial RNA cutoff was selected after inspection of the QC‐metric distributions in our clinical tumor samples to balance removal of clearly low‐quality cells against excessive exclusion of viable cells from freshly dissociated human tumor tissues. Cells with elevated mitochondrial RNA content were excluded during preprocessing to reduce the impact of low‐quality and stress‐enriched cells on downstream analyses. Data were normalized by NormalizeData (log‐normalization), and 5000 highly variable genes were identified with FindVariableFeatures.

After ScaleData and RunPCA, cross‐batch integration (institutional cohort + public cohorts) used Harmony v1.2.0 with default diversity penalty (theta = 2) and 30 PCs. This integration step was used to reduce technical heterogeneity across datasets, including pre‐analytical variation associated with cohort source. Graph‐based clustering employed FindNeighbors/FindClusters (resolution 0.4–0.8 as indicated in Figure legends). Low‐dimensional visualization used RunTSNE. To assess potential batch effects after integration, tSNE plots were generated separately for public data and our in‐house single‐cell dataset, as well as for each individual sample. These plots allow visual inspection of cell distribution across responder and non‐responder groups. Putative doublets were removed by excluding clusters co‐expressing discordant lineage markers and by manual inspection of UMI/gene distributions.

### 2.4 Cell Type Annotation

Cell type annotations were assigned based on canonical markers and differential expression (DE) profiles using Seurat's FindAllMarkers function (Wilcoxon test; min.pct = 0.25; log_2_FC.threshold = 0.25). Major cell lineages and their respective markers were defined as follows:

Epithelial/Tumor: EPCAM, KRT18, KRT19

Fibroblast: DCN, COL1A1, COL1A2, COL3A1

Endothelial: PECAM1, VWF, PLVAP, RAMP2

CD8^+^ T: CD8A, CD8B

CD4^+^ T: CD4; Treg: CD4, FOXP3

NK: GNLY, KLRF1

Myeloid: LYZ, CD14, CD68

Mast: TPSAB1, TPSB2, CPA3

B cells: CD79A, CD79B, MS4A1; Plasma cells: MZB1, IGHG1/IGHA1

For visualization purposes, a descriptive semi‐quantitative summary of CD8^+^ T cell subset enrichment was generated by classifying the direction and relative magnitude of the Ro/e analysis for each annotated CD8^+^ T cell subset in responder and non‐responder tumors. Symbols indicate weak/intermediate (±), moderate (+), or strong (++) relative enrichment and were used only as a compact overview of subset distribution patterns.

**FIGURE 1:**
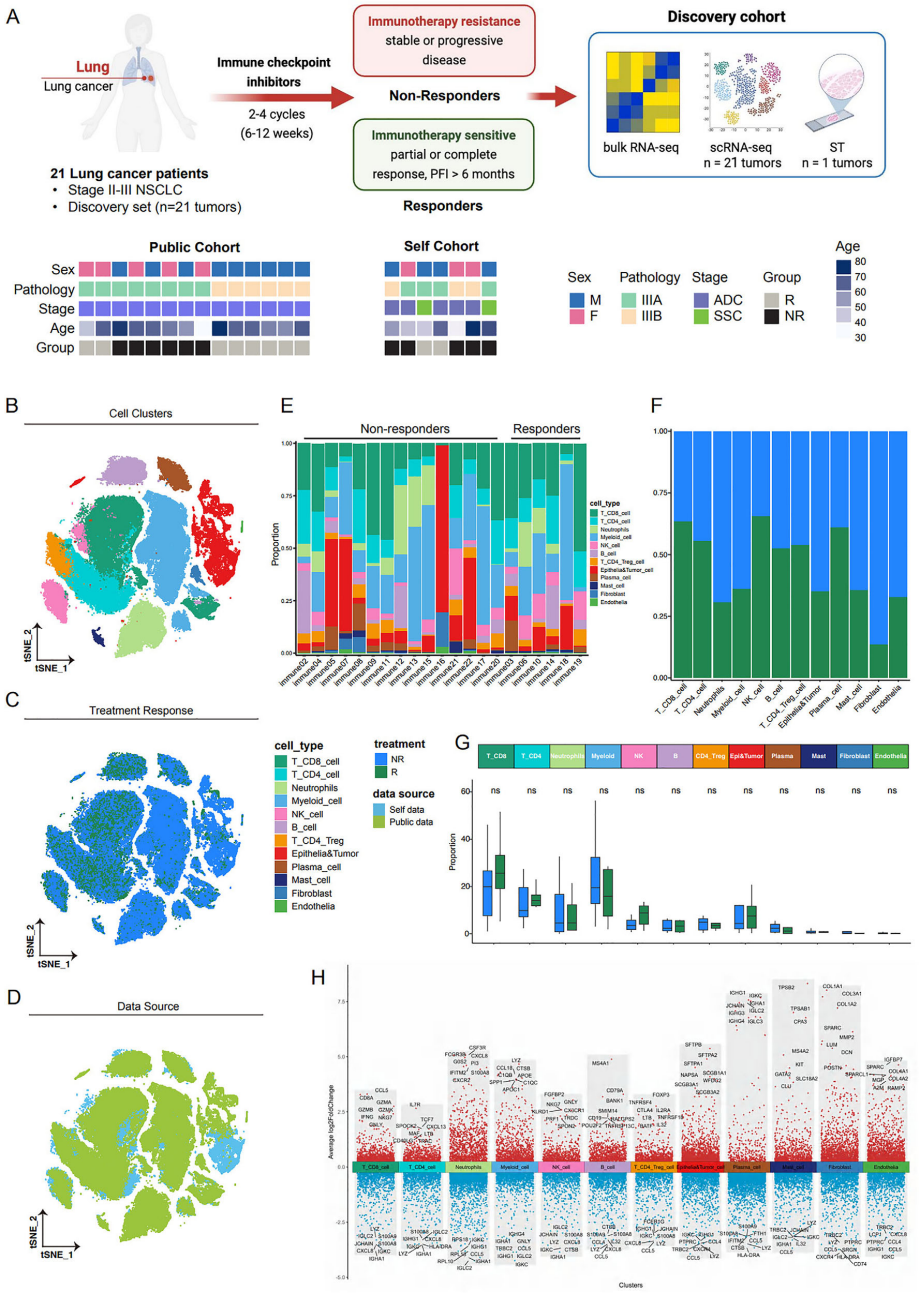
Study design and single‐cell landscape of ICI‐treated NSCLC (A) Schematic overview of the study design, cohort composition, and response definition. The integrated scRNA‐seq discovery atlas included NSCLC tumors from the in‐house cohort and publicly available ICI‐treated cohorts. One additional institutional non‐responder case underwent spatial transcriptomic profiling only and was used as an illustrative resistance‐focused spatial case study rather than for cross‐cohort statistical comparison. Patients were classified as responders (R) or non‐responders (NR) according to the clinical response criteria described in the Methods section. Heatmaps summarize the key clinicopathological characteristics of the in‐house and public cohorts. (B) t‐SNE of all single cells colored by major cell lineages identified after Harmony‐based integration and graph‐based clustering. (C) The same t‐SNE colored by treatment response (NR vs. R), showing the distribution of cells from the two clinical groups across the integrated atlas. (D) t‐SNE colored by data source, illustrating the mixing of in‐house and public datasets after Harmony integration. (E) Stacked bar plots showing per‐sample cell‐type composition, grouped by clinical response. (F) Overall cell‐type fraction per sample, displayed by response group as a descriptive overview of lineage distribution patterns. (G) Boxplots showing sample‐level comparison of the relative proportions of major cell populations between NR and R tumors. For each sample, the fraction of each lineage was calculated among all cells and compared between groups using the two‐sided Wilcoxon rank‐sum test. (H) Per‐cluster expression of canonical lineage‐specific marker genes across 11 major cell types, including epithelial/tumor, fibroblast, endothelial, myeloid, neutrophil, mast, B, plasma, NK, CD4^+^ T, and CD8^+^ T cells. Each row represents a gene and each column represents a cluster. Marker analysis confirms the identity of cell lineages defined by unsupervised clustering.

Cell type proportions were calculated per sample. Overall composition plots were used primarily for descriptive visualization. Where formal responder versus non‐responder comparisons were presented (Figure 1G), sample‐level proportions were compared using the two‐sided Wilcoxon rank‐sum test.

### 2.5 Inference of Malignancy and Genomic CNVs

To identify malignant epithelial cells, we used the inferCNV v1.12.0 tool, employing CD8^+^ T cells as the reference group. T cell populations have been widely used as non‐malignant internal reference controls for inferCNV‐based malignant‐cell identification in high‐quality single‐cell tumor studies [25, 26]. In our dataset, restricting the reference to the CD8^+^ T cell compartment provided a stable internal baseline for distinguishing large‐scale chromosomal copy‐number alterations in epithelial/tumor cells. Cells exhibiting large‐scale chromosomal arm‐level copy number variations (CNVs) were classified as malignant. CNV analysis was performed with default settings (cutoff = 0.1; denoise = TRUE; HMM = TRUE).

### 2.6 Differential Expression Analyses

Within each major lineage, differential expression analysis was performed using Seurat's FindMarkers function (v4.3.0) to compare gene expression between tumor subgroups. Analysis was conducted on normalized expression matrices. The default statistical test in FindMarkers, two‐sided Wilcoxon rank‐sum test was used to evaluate differences between groups, and Bonferroni correction was applied to adjust for multiple testing. All other cutoffs (minimum log‐fold change, percentage of expressing cells) were applied as in the original analysis. To reduce spurious signals from lowly detected genes, only genes expressed in at least 10% of cells in the tested group were considered. Differentially expressed genes (DEGs) were defined as genes with a fold change ≥ 1.2 and an adjusted P value ≤ 0.05. Full DEG lists are provided in Table S1D.

### 2.7 Cell–Cell Communication and Ligand–Receptor Inference

Cell‐cell communication analysis was performed using CellChat (v1.1.3). For each cell type, total incoming signaling strength was calculated as the sum of communication probabilities across all significant ligand–receptor interactions, weighted by ligand and receptor expression levels. Communication probabilities for each ligand–receptor pair were determined using CellChat's default method, which applies the law of mass action, and only interactions with *p* < 0.05 in permutation tests were retained. Heatmaps of incoming and outgoing signaling were generated with cell types aligned and colored consistently across responder and non‐responder groups, to allow direct visual comparison. Signaling pathways displayed in Figure 4 were selected based on statistical significance (*p* < 0.05) and biological relevance. Consensus ligand‐receptor pairs were also inferred using LIANA (v0.1.x) integrating multiple models (CellPhoneDB/Connectome/Logit) for cross‐validation purposes.

### 2.8 Monocle2 Pseudotime and Trajectory Analysis

We performed pseudotime and trajectory analysis using Monocle2 (version 2.28.0) to reconstruct the potential state transitions of selected cell populations, with a particular focus on the CD8^+^ T cell compartment in responder and non‐responder tumors. A new Monocle object was created from transcript count data using the newCellDataSet function. Differences in mRNA recovery across cells were normalized using the estimateSizeFactors and estimateDispersions functions. Feature genes used for trajectory inference were selected as genes expressed in at least 10% of cells in the analyzed dataset and showing significant variation by differentialGeneTest (*p* < 0.01). Dimensional reduction was then performed using the DDRTree algorithm implemented in the reduceDimension function, and cells were ordered along the inferred trajectory using the orderCells function.

Pseudotime‐dependent genes were identified using differentialGeneTest with fullModelFormulaStr = ∼ sm.ns(Pseudotime). The resulting expression dynamics along pseudotime were visualized using the plot_pseudotime_heatmap function. For the CD8^+^ T cell analysis, the inferred trajectory was visualized by pseudotime, trajectory state, treatment group, and annotated CD8^+^ T cell subset, and subset distributions along pseudotime were summarized using density plots.

### 2.9 Pathway and Functional Enrichment Analysis

DEGs identified from the single‐cell and bulk transcriptomic analyses were subjected to pathway enrichment analyses to determine biological processes enriched in each comparison using Seurat's FindMarkers function with two‐sided Wilcoxon rank‐sum test and Bonferroni correction. Enrichment was performed in R (v4.2.3) using the clusterProfiler (v4.8.1) package with Benjamini–Hochberg correction for multiple testing. The Gene Ontology (GO) Biological Process, Kyoto Encyclopedia of Genes and Genomes (KEGG), and MSigDB Hallmark gene‐set collections were used as references. Only terms with an adjusted *p* < 0.05 were considered significant.

### 2.10 Ferroptosis Signature Scoring

**FIGURE 2:**
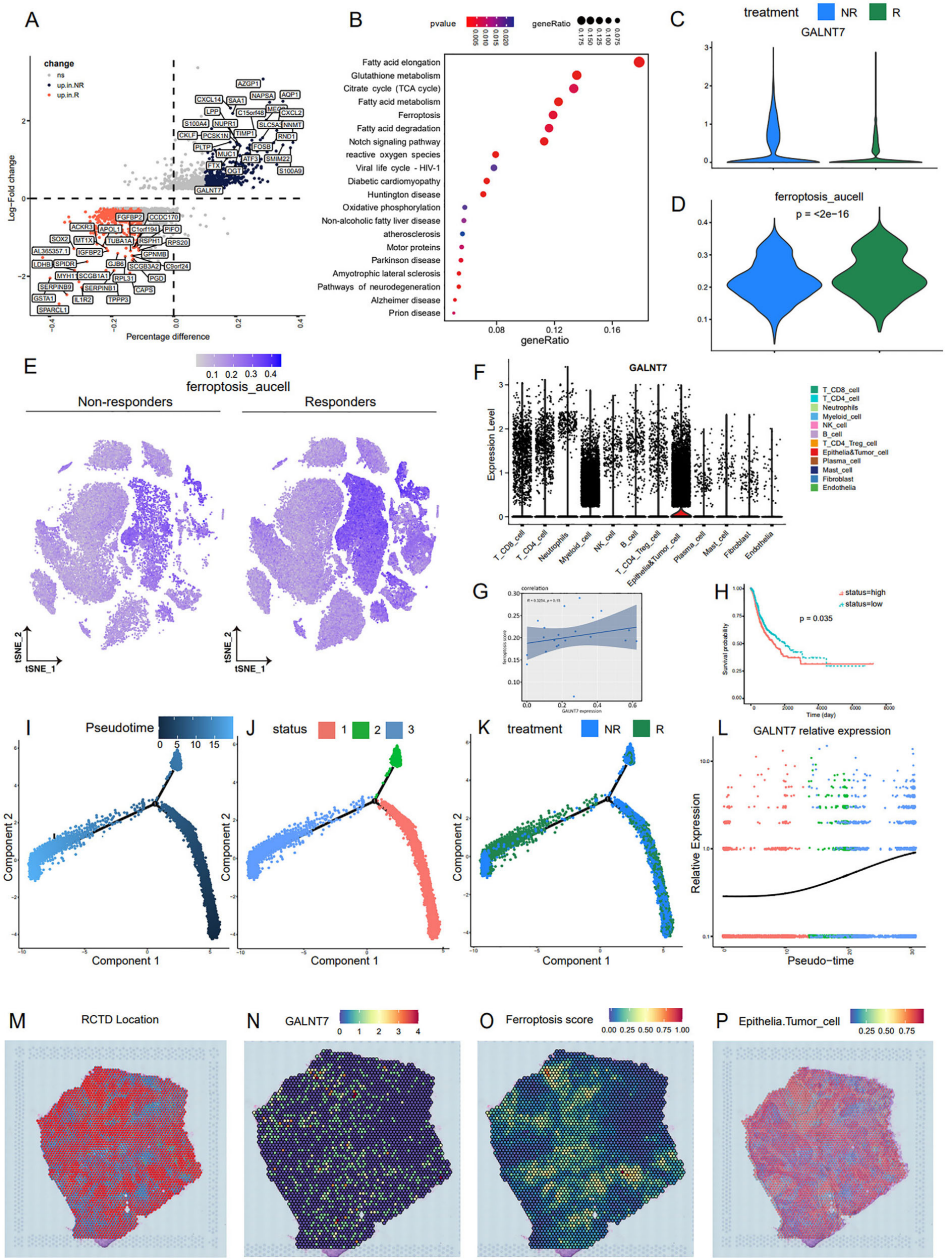
GALNT7 and ferroptosis pathway define resistant tumor cell states in NSCLC (A) Volcano plot showing DEGs between responders (R) and non‐responders (NR) tumor epithelial cells. (B) KEGG enrichment analysis of genes upregulated in R group. Ferroptosis, glutathione metabolism, and fatty acid elongation pathways were significantly enriched. (C,D) Violin plots comparing GALNT7 expression (C) and ferroptosis activity scores D between NR and R tumors, indicating higher GALNT7 in NR and higher ferroptosis activity in R. (E) t‐SNE visualization of ferroptosis activity across tumor epithelial cells from NR (left) and R (right) samples. (F) Violin plot showing GALNT7 expression across major cell populations, highlighting predominant enrichment in epithelial/tumor cells. (G) Donor‐level scatterplot showing the relationship between GALNT7 expression and ferroptosis AUCell score across epithelial/tumor‐cell samples. Each point represents one donor/sample, and the Spearman correlation coefficient and P value are shown in the panel. (H) Kaplan–Meier survival analysis of GALNT7 expression in the TCGA‐LUAD cohort, showing poorer OS in the GALNT7 high group. (I–K) Monocle2 trajectory analysis of epithelial/tumor cells. (I) Pseudotime visualization (J) branch differentiation (K) distribution by treatment group (NR vs. R). (L) Dynamic expression of GALNT7 along pseudotime, showing progressive upregulation toward NR states. (M–P) Spatial transcriptomic profiling of a single clinically distinct non‐responder case. (M) Spatial grid showing tissue capture regions; (N) spatial map of GALNT7 expression; (O) ferroptosis activity map; (P) epithelial/tumor‐cell distribution. These maps are shown as illustrative spatial observations and were not used for cohort‐level statistical inference.

A ferroptosis gene signature was curated from MSigDB (KEGG_FERROPTOSIS and related Hallmark pathways) and supplemented with canonical regulators reported in the literature, including SLC7A11, GPX4, ACSL4, and ALOX15. At the single‐cell level, ferroptosis activity was quantified using the AUCell (v1.20.1) algorithm implemented in R. AUCell calculates the area under the recovery curve (AUC) for each cell based on the ranked expression of the ferroptosis gene set (aucMaxRank = 0.05 × nFeatures). The AUC values represent the relative activity of the pathway per cell. For the cell‐level comparisons shown in Figure 2C,D, differences between responder and non‐responder epithelial/tumor cells were evaluated using a two‐sided Wilcoxon rank‐sum test at the single‐cell level.

At the bulk and TCGA levels, ferroptosis scores for each sample were estimated using GSVA/gene set enrichment analysis (GSEA) (method = “ssgsea”, tau = 0.25). Samples were stratified into high‐ and low‐score groups (median split), and associations with survival outcomes were analyzed using Cox regression and Kaplan–Meier curves (P values adjusted via Benjamini–Hochberg). Visualization of ferroptosis activity was performed using FeaturePlot, VlnPlot for single‐cell data and box or forest plots for bulk and clinical datasets.

For donor‐level correlation analysis, average GALNT7 expression and average ferroptosis AUCell score were calculated per sample within epithelial/tumor cells, and sample‐level associations were assessed using Spearman correlation. In addition, donor‐level correlations between GALNT7 and selected representative ferroptosis‐related genes were evaluated as supportive analyses, including the ferroptosis suppressors AIFM2/FSP1 and GCH1, and the ferroptosis driver LPCAT3.

### 2.11 Bulk RNA‐Sequencing (RNA‐seq)

Bulk RNA sequencing was performed on frozen tumors derived from shGALNT7‐LLC and shNT‐LLC groups (n = 3 per group; independent biological replicates). Detailed RNA extraction, library preparation, sequencing, differential‐expression analysis, and pathway‐enrichment procedures are described in Section 2.22

### 2.12 Public Cohort Validation (TCGA)

For external validation in an immunotherapy‐treated clinical cohort, bulk transcriptomic data from the OAK NSCLC study were analyzed after stratification into responder (R) and non‐responder (NR) groups according to the available clinical response annotation. Differential gene expression analysis between R and NR samples was performed using limma, with significance defined by adjusted *p* < 0.05 and |log2FC| > 0.5. To estimate differences in tissue‐level cellular composition, transcriptome deconvolution was performed using CIBERSORT, and inferred cell proportions were compared between R and NR groups using the Wilcoxon rank‐sum test. These results were used as an orthogonal validation of the single‐cell findings.

TCGA‐LUAD data (UCSC Xena) were further used to validate associations among GALNT7, ferroptosis signatures, and clinical outcome. Patients were dichotomized by median GALNT7 expression (high vs. low) (Table S1E). Survival analyses (OS/DFS where available) used survival v3.5‐7 and survminer v0.4.9 (univariate Cox; Kaplan‐Meier; log‐rank). Single‐sample pathway scores (including ferroptosis‐related antioxidant and lipid peroxidation modules) were computed by GSVA/ssGSEA v1.46.0 where indicated.

### 2.13 Cell Lines, Authentication, Culture, and Reagents

Human LUAD cell lines. A549 (human lung adenocarcinoma cell line) (ATCC CCL‐185) and Calu‐3 (human lung adenocarcinoma cell line)(ATCC HTB‐55) were obtained from ATCC. Cell identity was verified by STR profiling (Promega GenePrint 24) on receipt and every 6 months. Mycoplasma negativity was confirmed monthly by quantitative reverse‐transcription PCR (PCR) (LookOut Mycoplasma Detection Kit, Sigma MP0035). Cells were cultured in RPMI‐1640 (cell culture medium) (Gibco 11875093) supplemented with 10% heat‐inactivated fetal bovine serum (FBS) (Gibco A31608‐02) and 1% penicillin/streptomycin (Gibco 15140122) at 37 °C in 5% CO_2_. Cells were passaged at 70%–80% confluence and used within passages 4–12, with routine morphological checks. Ferroptosis modulators and solvents. Erastin (10 µM; Selleck S7242; 10 mM DMSO stock) was used to induce ferroptosis. Ferrostatin‐1 (Fer‐1) (ferroptosis inhibitor; 2 µM; Sigma SML0583) inhibited lipid peroxidation. To ensure iron availability, cultures were switched to FBS‐free RPMI‐1640 containing FeCl_3_ (5 µM) as indicated. Final DMSO ≤0.1% (v/v) in all conditions including vehicle.

Mitochondrial reactive oxygen species (ROS) scavenger (used only as rescue in lipid peroxidation assays). MitoTEMPO (mitochondria‐targeted antioxidant; Sigma‐Aldrich, SML0737) was used at 10 µM as a mitochondrial ROS scavenger in lipid‐peroxidation rescue experiments; matched solvent was used for controls.

General handling/QC. Experiments used independent biological replicates (*n* ≥ 3 unless stated). Reagents were aliquoted and single‐thawed (−20°C/−80°C). Instruments were calibrated daily; pipettes quarterly.

### 2.14 Animal Models, Husbandry, Randomization, and Ethics

*Cells and vectors*: Lewis lung carcinoma (LLC; ATCC CRL‐1642 STR‐profiled; mycoplasma‐tested monthly) were transduced with lentiviral shGALNT7 (short‐hairpin RNA targeting GALNT7) (sequence CCGGGCCTGAACTGTTCAACATCAT) or non‐targeting shNT (non‐targeting short‐hairpin control) in pLKO.1‐Puro/Hygro (Sigma MISSION) (Table S2A). Viral titer ≥1 × 10^8^ IU mL^−1^. Transductions (polybrene 8 µg mL^−1^) were followed at 48 h by antibiotic selection with puromycin (2 µg mL^−1^, 72 h; maintenance 1 µg mL^−1^) or hygromycin B (200 µg mL^−1^, 5–7 days; maintenance 100 µg mL^−1^), according to the resistance cassette.

*Mice and housing*: Male C57BL/6J mice (6–8 weeks; GemPharmatech, Strain NO. N000013) were housed SPF mice under a 12‐h light–dark cycle, 20–22°C, 40%–70% humidity, irradiated chow and acidified water ad libitum. Mice were co‐housed prior to inoculation and group‐assigned afterward. Health was monitored daily.

*Tumor establishment and randomization*: Subcutaneous tumors were established by injecting 1 × 10^6^ shGALNT7‐LLC or shNT‐LLC in PBS:Matrigel (1:1; 100 µL) into the flank (*n* = 6/group unless specified). Mice were enrolled and randomized at ≈80 mm^3^. Tumor volume was measured twice weekly (V = 0.5 × L × W^2^) by personnel unblinded to allocation. Body‐weight was recorded twice weekly.

*Humane endpoints and ethics*: Prespecified humane endpoints included tumor volume reaching 1500 mm^3^, tumor diameter ≥1.5 cm, ulceration, or ≥20% body‐weight loss. Mice were monitored regularly throughout the experiment, and euthanasia was performed once humane endpoints were reached or before tumor burden exceeded the permitted limits. All animal procedures were reviewed and approved by the Laboratory Animal Ethics Committee of West China Hospital, Sichuan University (IACUC), protocol 20240923015, and complied with ARRIVE guidelines.

*Tissue processing*: Tumors were harvested 14–21 days post‐inoculation (diameter 8–12 mm) and processed within 1 h to single‐cell suspensions (viability >85% by trypan blue). Portions were snap‐frozen for protein/RNA and fixed for FFPE/TEM.

### 2.15 SiRNA (Small Interfering RNA)‐Mediated GALNT7 Knockdown in Human LUAD Cells

*Design and transfection*: Three siRNAs against human GALNT7 were used: siGALNT7‐1 (5′‐GGAUAAUGACCUCUCUGUtt‐3′), siGALNT7‐2 (5′‐GGUUAAGUGACACUCCUAtt‐3′), siGALNT7‐3 (5′‐GGGAAGUGGACAGACGAUtt‐3′) (Table S2A); non‐targeting siRNA served as control. A549/Calu‐3 were seeded to ≈50%–60% confluence. siRNA (50 nM) was transfected with Lipofectamine RNAiMAX (Lipofectamine RNAiMAX (transfection reagent)) (Thermo 13778075) in Opti‐MEM; media were replaced 6 h later.

*Validation and basic readouts*: At 48–72 h, knockdown was verified by qRT‐PCR (PrimeScript RT, Takara RR037A; ΔΔCt vs. ACTB/GAPDH) and immunoblotting (§2.5). Viability at 72 h was assessed by CCK‐8 (cell viability assay) (Dojindo, CK04); DNA synthesis by Click‐iT EdU (DNA synthesis labeling assay) Imaging Kit, Alexa Fluor 488 (Invitrogen/Thermo Fisher, C10337) with fixed imaging parameters. Apoptosis was quantified by Annexin V‐FITC Apoptosis Detection Kit with PI (BD Pharmingen, 556547), acquiring ≥30 000 events/sample on BD FACSymphony A3; FlowJo v10.8.1 for analysis.

Unless otherwise specified, the downstream functional assays were performed using siGALNT7‐1.

### 2.16 Ferroptosis Readouts and Lipid Peroxidation Assays

*Experimental scheme*: To interrogate lipid peroxidation, A549 and Calu‐3 cells transfected with siNT (control) or siGALNT7 were tested ± MitoTEMPO (10 µM). Where indicated, erastin (10 µM) was used to accentuate stress; Fer‐1 (2 µM) served as antagonists. Time windows and solvent (DMSO ≤0.1%) were matched across groups.

*Oxidized BODIPY fluorescence*: Cells were incubated with Oxidized BODIPY (Thermo Fisher Scientific, Image‐iT Lipid Peroxidation Kit, C10445) at 2 µM for 30 min, 37°C, washed, and analyzed by flow cytometry. Oxidized fluorescence was collected in FITC; when applicable, the reduced counterpart was recorded in PE. Oxidized BODIPY MFI and/or oxidized/reduced ratio per cell was reported. Unstained and positive controls (e.g., cumene hydroperoxide) set voltages and gates.

*LPO level (%)*: LPO (%) was defined as the percentage of Oxidized‐BODIPY–positive cells above a prespecified gate among total live cells. Gates were fixed across experiments using the control distribution. Results are reported for Control (siNT), siGALNT7, and siGALNT7 + MitoTEMPO in A549 and Calu‐3.

*QC and statistics*: For each condition, ≥3 biological replicates and ≥30 000 live events/replicate were acquired. Settings (PMT voltages, thresholds) were locked after standardization. Data were analyzed in FlowJo v10.8.1 and plotted in GraphPad Prism v10.6.1 using unpaired two‐tailed t‐test (two groups) or one‐way ANOVA with Tukey's multiple‐comparisons test (≥3 groups), as specified in §2.24.

### 2.17 Immunoblotting

Cells/tumors were lysed in ice‐cold RIPA supplemented with Halt Protease and Phosphatase Inhibitor Cocktail (100×, Thermo Fisher Scientific; 78440). Lysates were clarified (14 000 g, 15 min, 4°C). Protein was quantified by Pierce BCA Protein Assay Kit (Thermo Fisher Scientific; 23225). Equal protein (20–40 µg) was separated by SDS‐PAGE (10%–12%) and transferred to PVDF (0.45 µm). After blocking (5% BSA, 1 h, RT), membranes were incubated with primary antibodies (1:1000 unless noted): GALNT7 (Abcam ab254971), SLC7A11 (Abcam ab307601), glutathione peroxidase 4 (GPX4) (Abcam ab125066), acyl‐CoA synthetase long‐chain family member 4 (ACSL4) (Proteintech 22401‐1‐AP), β‐Actin (Sigma–Aldrich, A5441; clone AC‐15). HRP secondaries: anti‐rabbit IgG (CST 7074; 1:5,000) and anti‐mouse IgG (CST 7076; 1:5000) for 1 h; ECL imaging on LI‐COR Odyssey CLx; ImageStudio v5.2 for densitometry normalized to β‐Actin (Table S2B).

### 2.18 Transmission Electron Microscopy (TEM)

Specimens were fixed in 2.5% glutaraldehyde + 2% paraformaldehyde (0.1 M cacodylate, pH 7.4) for 24 h at 4°C, post‐fixed in 1% OsO_4_ for 1 h, dehydrated (graded ethanol), and embedded in Epon 812. Ultrathin sections (≈70 nm) were cut, mounted on copper grids, and post‐stained with 2% uranyl acetate (10 min) and Reynolds’ lead citrate (5 min) at room temperature. Images were acquired on a Hitachi HT7800 at 80 kV.

TEM is presented as representative micrographs comparing GALNT7‐silenced and control conditions; no morphometric quantification was performed. Acquisition settings (magnification, exposure) were kept constant across groups.

### 2.19 Immunological Profiling (Flow Cytometry of TILs; IHC and IF on FFPE)

*Tumor‐infiltrating lymphocytes (TILs)*: Fresh tumors were weighed, minced on ice, and digested in Collagenase IV (1 mg mL^−1^) + DNase I (0.1 mg mL^−1^) prepared in RPMI‐1640 for 2 h at 37 °C with orbital rotation. Digests were passed through 70‐µm strainers, RBC were lysed (ACK, 1–2 min, RT), and cells were washed twice in FACS buffer (PBS + 2% FBS + 2 mM EDTA, ice‐cold). Cell counts and viability were determined by trypan blue; samples with viability < 80% were excluded a priori. For all downstream staining, cells were kept on ice and protected from light. Technical replicates were not pooled; per‐sample metadata (tumor weight, viability, live‐event yield) were recorded for normalization.

*Flow cytometry*: Cells were Fc‐blocked with TruStain FcX (anti‐mouse CD16/32), clone 93 (BioLegend, 101320) for 10 min on ice. Tumor‐infiltrating leukocytes were analyzed for CD45, CD3, CD8, and IFN‐γ expression; dead cells were excluded using Zombie NIR Fixable Viability Dye (BioLegend, 423105). CountBright Absolute Counting Beads (Thermo Fisher, C36950) were added immediately before acquisition; absolute counts were calculated as cells/µL = (cell events/bead events) × bead concentration and reported as cells per gram of tumor using recorded tumor weights. Data were acquired on a BD FACSymphony A3 with standardized PMT voltages; UltraComp eBeads (Invitrogen, 01‐2222‐41) were used each run for compensation (Table S2B). At least 30 000 live events/sample were collected, with FMO controls (notably IFN‐γ FMO) plus unstained/isotype controls to define thresholds; pre‐specified exclusions included carryover, clogs, <20 000 live events, or aberrant negatives. Events were gated sequentially as singlets (FSC‐A vs. FSC‐H), viable cells (Zombie NIR negative), CD45^+^ leukocytes, and CD3^+^CD8^+^ T cells; within CD3^+^CD8^+^, IFN‐γ^+^ cells were quantified using FMO‐defined thresholds. Primary outcomes were frequency (%) and absolute counts of CD3^+^CD8^+^ and IFN‐γ^+^CD8^+^ (normalized as cells/g tumor). Analyses were performed in FlowJo v10.8.1 with identical gates batch‐applied across samples; statistics followed Section 2.20 (GraphPad Prism v10.6.1; R v4.5.1)

*Immunohistochemistry (IHC) on FFPE*: For the immunohistochemical analyses shown in Figure 7D, FFPE tumor sections were evaluated for Ki‐67, PD‐L1, PD‐1, and CD8 staining. The corresponding staining signals were quantified as integrated optical density (IOD) for group comparison. During post‐publication verification, the exact historical antibody product identifiers and detailed reagent metadata for these IHC assays could not be reconstructed with sufficient confidence; therefore, unverified catalog numbers or antibody products have not been retrospectively assigned.

Immunofluorescence (IF) on FFPE (conventional indirect immunofluorescence). Consecutive 4‐µm FFPE tumor sections were deparaffinized, rehydrated, and subjected to heat‐induced epitope retrieval in 10 mM sodium citrate buffer (pH 6.0, 95–98°C, 20 min), then cooled to room temperature. Sections were permeabilized with 0.1% Triton X‐100 (5 min) and blocked in 5% BSA + 10% normal goat serum (30min, RT). Primary antibodies against CD8 and IFN‐γ were applied overnight at 4°C; nuclei were counterstained with DAPI (300 nM, 5 min). Species‐appropriate secondaries (goat anti‐mouse Alexa Fluor 488, 1:500; goat anti‐rabbit Alexa Fluor 594, 1:500; Invitrogen/Thermo Fisher) were incubated 1 h at RT in the dark (Table S2B). Slides were mounted with ProLong Glass Antifade Mountant (Thermo Fisher) and cured ≥24 h before imaging. Whole‐slide fluorescence images were acquired on a Zeiss Axio Scan.Z1 using a Plan‐Apochromat 20×/0.8 NA objective (whole slide) and 40×/0.95 NA for high‐magnification Merge composites and Enlarge insets; exposure, gain, and illumination were fixed across samples. Quantification was performed in HALO (Indica Labs) v3.x with the HighPlex FL module, using thresholds derived from negative/isotype and single‐stain controls and then batch‐applied to the cohort. Regions of interest excluded necrosis and artifacts a priori. Outputs included CD8^+^ cell density (cells/mm^2^), IFN‐γ^+^ cell density (cells/mm^2^), and—where indicated—IFN‐γ^+^ area fraction (%) within tumor region of interest (ROIs). Analyses were performed blinded to group allocation and exported as CSV for statistics.

### 2.20 In Vivo Therapeutic Studies (αPD‐1/Isotype, Fer‐1)

*Study arms and dosing*: At ≈80 mm^3^, mice were randomized (*n* = 6/group) to receive: i) anti‐PD‐1 (Bio X Cell BE0146, 10 mg kg^−1^, intratumoral, every 3 days on study days 0, 3, 6, 9, and 12); ii) isotype control (rat IgG2a, κ, clone 2A3; Bio X Cell BE0089, same route/schedule as anti‐PD‐1); and iii) Ferrostatin‐1 (Fer‐1) (5 mg kg^−1^, intraperitoneal, once daily). Combination cohorts included shGALNT7 ± Fer‐1 with matched vehicle and isotype controls.

*Endpoints (no survival analysis)*: Primary endpoints were tumor volume kinetics (calipers; V = 0.5 × L × W^2^) and terminal tumor weight at the experimental endpoint (day 15 post‐randomization for the treatment study shown in Figure 8) or earlier if humane endpoints were met. Tolerability was monitored by body‐weight and clinical observation.

### 2.21 In Vitro Functional Assays

*Apoptosis (A549, Calu‐3)*: Annexin V‐FITC/PI staining (Annexin V‐FITC Apoptosis Detection Kit with PI; BD Pharmingen, 556547) at 48–72 h post‐transfection; ≥30 000 events/sample; BD FACSymphony A3 acquisition; FlowJo v10.8.1 analysis. Total apoptotic fractions were quantified as Annexin V‐positive cells, including early and late apoptotic populations. Staurosporine (1 µM, 4 h) served as the positive control in assay qualification.

Lipid peroxidation endpoints. For each cell line and condition (Control/siGALNT7/siGALNT7+MitoTEMPO;±erastin/Fer‐1 where applicable), we reported Oxidized BODIPY MFI or ratio and LPO(%).

### 2.22 Bulk RNA‐seq of Mouse Tumors (sh‐GALNT7 vs. Control; 3 vs. 3)

*Cohorts & extraction*: Frozen tumors from sh‐GALNT7 (*n* = 3; Knock Down‐1/2/3) and shNT control (*n* = 3; Control‐1/2/3) were processed for RNA. TRIzol extraction with on‐column DNase; Bioanalyzer RIN ≥ 7; Qubit (fluorometric quantification platform) quantification.

*Library & sequencing*: Poly(A)‐selected, strand‐specific libraries were prepared with the NEBNext Ultra II Directional RNA Library Prep Kit for Illumina (NEB, E7760/E7765; 400–500 ng input) and sequenced on an Illumina NovaSeq (2 × 150 bp) to a depth of ≥30 million read pairs per sample. Processing & QC. Reads were trimmed and quality‐filtered with fastp (read trimming/quality control software), then aligned to GRCm39 using STAR (—twopassMode Basic). Gene‐level counts were generated by featureCounts (Subread) against GENCODE mouse release M38 (GRCm39; Ensembl release 115) annotation. Run‐level summaries were aggregated with MultiQC v1.31.

*Differential expression*: Raw count matrices were analyzed in DESeq2 v1.48.2 (Bioconductor 3.21). Genes with low counts were filtered (rowSums ≥ 10 retained). The design formula was ∼ group (sh‐GALNT7 vs. shNT). Log_2_ fold changes were shrinkage‐estimated with apeglm. Significance was defined as FDR (Benjamini–Hochberg) q < 0.05 with |log_2_FC| ≥ 1.0 (stringent threshold to emphasize biologically meaningful changes). Volcano plots were generated in ggplot2 v4.0.0. Bioconductor+1

Pathway enrichment (five collections). Ranked gene lists (by Wald statistic; equivalent results obtained using signed −log_10_P × sign(log_2_FC)) were subjected to preranked GSEA using clusterProfiler v4.16.0 and fgsea v1.34.2. Gene‐set resources included: MSigDB HALLMARK, Gene Ontology (BP/MF/CC; Mouse MSigDB v2025.1. Mm, June 2025 release), KEGG (mmu), Reactome, and WikiPathways (mouse). Parameters: nPerm = 10 000, minSize = 15, maxSize = 500; significance FDR q < 0.05. Outputs for each collection included ID, Description, NES, p.adj, core‐enrichment genes, Direction (Control↑ / KD↑) and were organized as Table S3 (HALLMARK/GO/KEGG/Reactome/WikiPathways). GSEA MSigDB+3Bioconductor+3Bioconductor+3

*Data availability*: The raw and processed RNA‐seq data and analysis scripts are not publicly deposited at this time but are available from the corresponding author upon reasonable request (email: guiyi01@wchscu.cn).

### 2.23 Tumor Size and Weight Measurements

Tumor length (L, longest axis) and width (W, longest perpendicular) were measured with digital calipers; volume was calculated as V = 0.5 × L × W^2^. Measurements were performed at consistent times of day (morning, ZT2–ZT4) to minimize diurnal variation. For each mouse, two consecutive readings per dimension were taken; discrepant reads (>10%) triggered a third measurement, with the median recorded. Calipers were zeroed before each session and calibrated weekly against gauge blocks. Mice were gently restrained without anesthesia; any tumors with ulceration or irregular geometry were flagged and measured along two orthogonal widths, with notes retained for sensitivity analyses. At endpoint or humane termination, tumors were excised, gently blotted, and weighed on an analytical balance (±0.1 mg). Body weight was recorded twice weekly (e.g., Monday and Thursday) on the same scale prior to dosing.

### 2.24 Statistics and Reproducibility

Unless otherwise specified, data are presented as mean ± SEM. For two‐group comparisons, unpaired two‐tailed Student's t‐test were used for normally distributed data, whereas Mann–Whitney U test were used when normality assumptions were not met. For comparisons involving three or more groups, one‐way ANOVA followed by Tukey's multiple‐comparisons test was used for normally distributed data, whereas Kruskal–Wallis test followed by Dunn's post hoc test were used for non‐normally distributed data.

Normality was assessed using the Shapiro–Wilk test, and homogeneity of variance was evaluated using Levene's test where applicable. For sample‐level cell‐type proportions presented in Figure 1G, responder and non‐responder groups were compared using the two‐sided Wilcoxon rank‐sum test. Other single‐cell comparisons were performed as described in the corresponding Methods subsections. Differential expression analyses for single‐cell subsets were performed using the methods described in the corresponding Methods sections, with multiple‐testing correction applied to the resulting P values where appropriate. For pathway enrichment and other high‐dimensional analyses, multiple testing was controlled using the Benjamini–Hochberg false discovery rate (FDR) method where applicable.

Unless otherwise stated, a two‐sided *p* value < 0.05 was considered statistically significant. The statistical test used for each analysis is indicated in the corresponding Methods subsection, figure legend, or both. All statistical analyses were performed using GraphPad Prism v10.6.1 and R v4.5.1.

## 3 Results

### 3.1 Single‐Cell Atlas of Immunotherapy–Treated NSCLC and Overall Landscape

To delineate the cellular programs associated with clinical response to immune checkpoint inhibitors (ICIs) in NSCLC, we generated an integrated single‐cell transcriptomic discovery atlas combining treatment‐naïve in‐house NSCLC tumors profiled by 10x Genomics scRNA‐seq with publicly available ICI‐treated NSCLC dataset (Figure 1A). In addition, one independent institutional non‐responder case was analyzed separately by spatial transcriptomics as an illustrative resistance‐focused case study and was not included in the integrated scRNA‐seq cohort or in cross‐cohort statistical analyses. Patients in the integrated scRNA‐seq atlas were categorized as responders or non‐responders according to the predefined clinical response criteria described in the Methods section.

Following stringent quality control and Harmony integration (Figure S1A), a total of 24 361 cells of R group and 77 188 cells of NR group passed filtering and were subjected to downstream analyses (Table S4A–C). Because the integrated human dataset combined institutional and public ICI‐treated cohorts with different clinical contexts, we interpreted this dataset primarily as a cross‐cohort discovery atlas for identifying shared resistance‐associated cellular states rather than as a fully balanced clinical comparison cohort. Unsupervised clustering resolved 11 major cell lineages, including epithelial/tumor, fibroblast, endothelial, myeloid, neutrophil, mast, B, plasma, NK, CD4^+^ T, and CD8^+^ T cells (Figure 1B,H and Figure S1B–D). Marker analysis confirmed canonical lineage‐specific genes across clusters (Figure 1H and Figure S2). To further examine batch effects and sample‐level heterogeneity, we separated tSNE plots by data source (public *vs*. in‐house) and by individual patient samples (Figure S1E–G). These plots confirm minimal batch effects across cohorts and illustrate the distribution of R and NR cells across different datasets. Overall, T cells constituted the largest immune population, comprising 23,119 CD8^+^ cells (29.9%), 13,204 CD4^+^ cells (17.1%), and 4357 Treg cells (5.6%), followed by myeloid cells (22 568; 29.3%), epithelial/tumor cells (11 624; 15.1%), and NK cells (5353; 7.0%) (Table S4C).

Across individual samples, marked heterogeneity in immune composition was observed (Table S4D). The proportion of CD8^+^ T cells ranged from 1.0% (P16) to 51.6% (P21), while myeloid cells accounted for 13.6%–64.8% and NK cells for 0.7–13.5% of total cells, underscoring substantial inter‐patient variability.

Cells from in‐house and public datasets co‐localized on the t‐distributed stochastic neighbor embedding (t‐SNE), confirming minimal batch effects after integration (Figure 1C,D). Responder and non‐responder tumors exhibit similar overall lineage distributions across the integrated atlas and per‐sample composition profiles (Figure 1E–G and Table S4B). Minor variations are observed descriptively, but no statistically significant differences are detected across samples, consistent with the compositional constraints of cell type proportions. Figure 1F is presented as a descriptive overview of lineage distribution patterns after normalization within each response group and was not intended as a direct comparison of absolute cell counts. Formal sample‐level statistical comparison was performed in Figure 1G, in which the proportion of each major immune lineage among all immune cells was calculated for each specimen and compared between responder and non‐responder groups using the two‐sided Wilcoxon rank‐sum test. After reanalysis, broad immune‐lineage proportions showed only limited separation between groups, and non‐significant comparisons are indicated as “ns”.

### 3.2 GALNT7 and Ferroptosis Signature Define Tumor Cell States Associated with Immunotherapy Response

To explore tumor‐intrinsic mechanisms underlying differential immunotherapy response, we focused on the epithelial/tumor cell compartment and performed comparative transcriptional profiling between NR and R (Figure 2A).

CNV analysis confirmed the malignant identity of the epithelial clusters (Figure S3). Differential gene analysis revealed distinct transcriptional programs, with metabolic and ferroptosis‐related genes enriched in R tumors. KEGG enrichment of R‐upregulated genes highlighted pathways involved in fatty acid metabolism, glutathione metabolism, and ferroptosis (Figure 2B and Table S5A).

Hallmark and GO enrichment analyses further supported the metabolic divergence between R and NR epithelial cells (Figure S4 and Table S5B,C).

Specifically, R tumors showed enrichment of inflammatory and epithelial–mesenchymal transition–related programs, including TNFA signaling via NF‐κB, apoptosis, KRAS signaling up, and epithelial–mesenchymal transition (Figure S4A,B). In contrast, NR tumors were characterized by upregulation of oxidative and metabolic processes such as oxidative phosphorylation, fatty‐acid metabolism, hypoxia response, and tumor protein p53 pathway (Figure S4C,D), highlighting a ferroptosis‐prone metabolic state potentially associated with improved immunotherapy sensitivity. Integrative analysis of these datasets, together with previous reports, revealed that ferroptosis‐ and lipid metabolism‐related programs are closely linked with immunotherapy responsiveness, suggesting that metabolic control of ferroptotic vulnerability may underlie differential treatment outcomes.

Violin plot comparison showed that GALNT7 expression was significantly higher in NR tumors (Figure 2C), whereas ferroptosis scores were markedly elevated in R tumors (Figure 2D,E). Cell‐type–specific expression analysis showed that GALNT7 was predominantly enriched in epithelial/tumor cells, with comparatively lower expression in most immune or stromal populations (Figure 2F).

Consistent with this observation, TCGA analysis confirmed that high GALNT7 expression correlated with poorer overall survival (OS) in LUAD patients (Figure 2H).

Because cell‐level correlation may overestimate statistical significance by treating individual cells as independent observations, we re‐evaluated the association between GALNT7 expression and ferroptosis activity at the donor level within epithelial/tumor cells. Specifically, average GALNT7 expression and average ferroptosis AUCell score were aggregated per sample and assessed using Spearman correlation. Donor‐level analysis showed a positive trend between GALNT7 expression and ferroptosis score, although this association did not reach statistical significance (Figure 2G). We therefore present this result as a donor‐level trend rather than as definitive evidence of a strong association. To provide additional mechanistic context, donor‐level correlations between GALNT7 and three representative ferroptosis‐related genes—AIFM2/FSP1, GCH1, and LPCAT3—were also examined and are shown in Figure S5A as supportive analyses.

To further examine the association between GALNT7 expression and the tumor immune contexture, we conducted a pan‐cancer correlation analysis using TCGA datasets and multiple immune deconvolution algorithms. As shown in Figure S5B, GALNT7 exhibited predominantly negative correlations with diverse immune‐cell populations, particularly CD8^+^ T cells, NK cells, and cytotoxic immune signatures, especially evident in LUAD and LUSC. These findings suggest that elevated GALNT7 may contribute to an immunosuppressive TME, consistent with its role in ferroptosis resistance.

To dissect the dynamic transcriptional transitions associated with treatment response, we applied Monocle2 pseudotime analysis to tumor epithelial cells (Figure 2I–L). The trajectory inferred a gradual transcriptional evolution from responder‐like to non‐responder–like states, accompanied by a steady increase in GALNT7 expression along pseudotime (Figure 2L). In a single clinically distinct non‐responder case analyzed by spatial transcriptomics, GALNT7 transcripts were overall low in abundance and did not show statistically tested region‐specific enrichment at the current spot‐level resolution (Figure 2M–P). Ferroptosis activity and epithelial/tumor‐cell distribution showed visually heterogeneous spatial patterns. These data are therefore presented only as illustrative spatial context for a resistant tumor state, rather than as definitive or generalizable evidence. Collectively, these results suggest that GALNT7 upregulation and ferroptosis suppression characterize resistant tumor epithelial states in NSCLC, potentially contributing to immunotherapy resistance.

### 3.3 Immune Cell Landscape Associated with Immunotherapy Response

**FIGURE 3:**
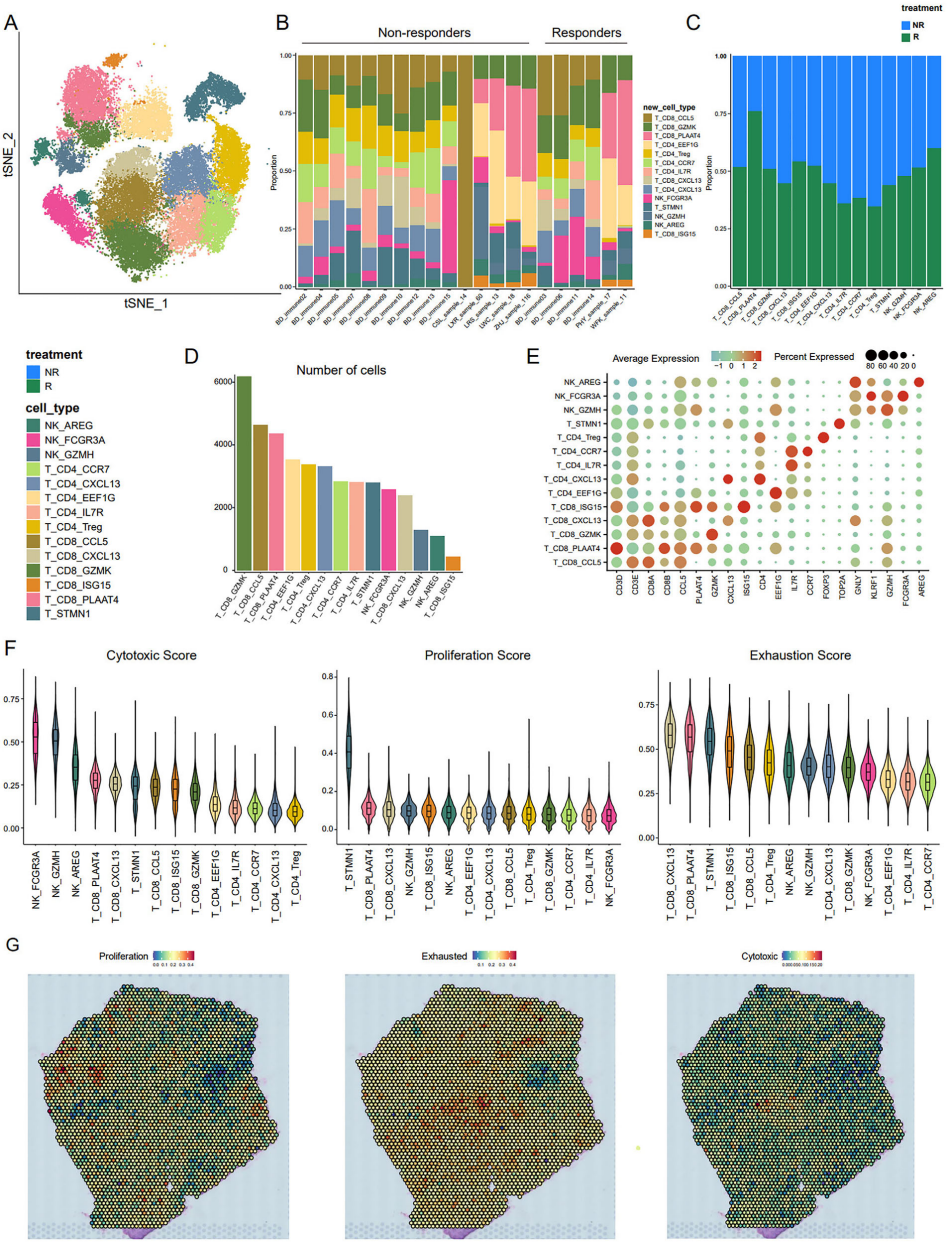
Single‐cell immune landscape in responders (R) and non‐responders (NR) to immunotherapy (A) t‐SNE visualization of immune‐lineage single cells from NSCLC tumors, colored by cell subtype. Thirteen clusters were identified. (B) Stacked bar plots showing cell‐type proportions per sample, stratified by treatment response (NR *vs*. R). (C) Overall abundance of major immune compartments in each response group. (D) Bar plot of the total number of cells identified per immune subset across all samples. (E) Dot plot displaying marker gene expression profiles defining NK and T cell subsets (average expression and percent of cells expressing each marker). (F) Immune cell subset stratification based on cytotoxicity, exhaustion, and proliferation scores. (G) Spatial maps of proliferation, exhaustion, and cytotoxicity scores in a single clinically distinct NR case. These panels are shown as descriptive spatial observations and were not used for formal spatial correlation testing or cohort‐level inference.

To characterize the immune microenvironment underlying treatment response, we next focused on TILs and natural killer (NK) cells derived from R and NR tumors. Unsupervised clustering and dimensional reduction of immune‐lineage cells revealed 13 distinct subpopulations, including NK (AREG^+^, FCGR3A^+^, GZMH^+^), CD4^+^ T (CCR7^+^, CXCL13^+^, IL7R^+^, EEF1G^+^, Treg), and CD8^+^ T (CCL5^+^, CXCL13^+^, GZMK^+^, ISG15^+^, PLAAT4^+^, STMN1^+^) subsets (Figure 3A). The clustering was well resolved, with distinct marker expression patterns defining each lineage. Quantification across samples showed that the relative composition of NK and T cell subsets varied across individuals and treatment responses (Figure 3B,C and Table S6A,B). Both R and NR tumors contained abundant CD8^+^ T cells, with heterogeneous distributions of effector (GZMK^+^, CCL5^+^) and exhausted‐like (CXCL13^+^, ISG15^+^) phenotypes. NK subtypes (AREG^+^, FCGR3A^+^) and regulatory T cells were also detected at moderate frequencies (Table S6C). The total number of cells within each immune subset is summarized in Figure 3D, highlighting the predominance of T‐cell–derived populations in the dataset. The dot plot analysis (Figure 3E) demonstrated canonical lineage‐specific gene signatures consistent with known NK and T‐cell programs. We next compared the proportional abundance of each immune subset between R and NR groups (Figure 3B,C). To provide orthogonal bulk‐level validation of these response‐associated differences, we further analyzed an external immunotherapy‐treated NSCLC bulk RNA cohort and compared responder and non‐responder samples at both the gene‐expression and inferred cell‐composition levels (Figure S5C,D). Bulk transcriptomic comparison revealed distinct response‐associated gene‐expression patterns between the two groups, while transcriptome deconvolution indicated differences in tissue‐level immune composition consistent with the overall trends observed in the single‐cell dataset. These bulk‐level findings support the presence of response‐associated transcriptional and immune‐compositional remodeling and are broadly consistent with the single‐cell analyses. Consistently, pan‐cancer bulk RNA‐seq analyses (e.g., TCGA) showed that higher GALNT7 expression correlated with lower CD8/T‐cell cytotoxic signatures, as summarized in Figure S5B. Although broad immune‐lineage proportions showed only limited separation between groups after sample‐level comparison, finer state‐level analysis revealed clearer response‐associated differences within the CD8^+^ T cell compartment. These findings support immune remodeling at the level of specific functional states rather than total lineage abundance alone.

We utilized a previously published dataset [27] to stratify immune cell subsets based on their cytotoxicity, exhaustion, and proliferation scores, which were derived from the expression of specific gene markers (Figure 3F). First, Cytotoxic scoring delineated a graded NK→T continuum in which CD16^+^/GZMH^+^ NK subsets consistently occupied the highest tier, whereas AREG^+^ NK cells showed attenuated cytotoxicity suggestive of tissue‐repair skewing; cytotoxic CD8 clusters (including PLAAT4^+^ and CXCL13^+^ states) ranked below peak NK effectors yet remained clearly effector‐competent [28]. Next, Exhaustion scoring reprioritized the landscape: CXCL13^+^/PLAAT4^+^ CD8^+^ subsets moved to the forefront, matching progenitor‐exhausted phenotypes that retain effector potential under chronic stimulation, while cytotoxic NK states shifted downward—indicating partial decoupling between cytotoxic capacity and exhaustion [29]. Finally, Proliferation scoring favored metabolically poised CD8 clusters (CXCL13^+^ and PLAAT4^+^) and cycling T cells, aligning with canonical cell‐cycle modules (AURKA/BUB1/CCND1), whereas terminal NK effectors generally displayed lower proliferative activity [30]. Taken together, this tri‐axis mapping reveals a coordinated yet partially independent architecture—highly cytotoxic NK peaks, proliferative/metabolically adapted CD8 programs, and exhaustion‐biased progenitors—that likely stratifies prognosis and immunotherapy responsiveness.

In the same illustrative spatial case, regions with relatively higher proliferation scores visually coincided with areas showing lower exhaustion and relatively higher cytotoxicity (Figure 3G). Because this analysis was limited to a single clinically distinct case and no formal spatial correlation testing was performed, these patterns are presented only as descriptive spatial observations rather than as statistically conclusive evidence.

### 3.4 CD8 T Cell Landscape Associated with Immunotherapy Response

**FIGURE 4:**
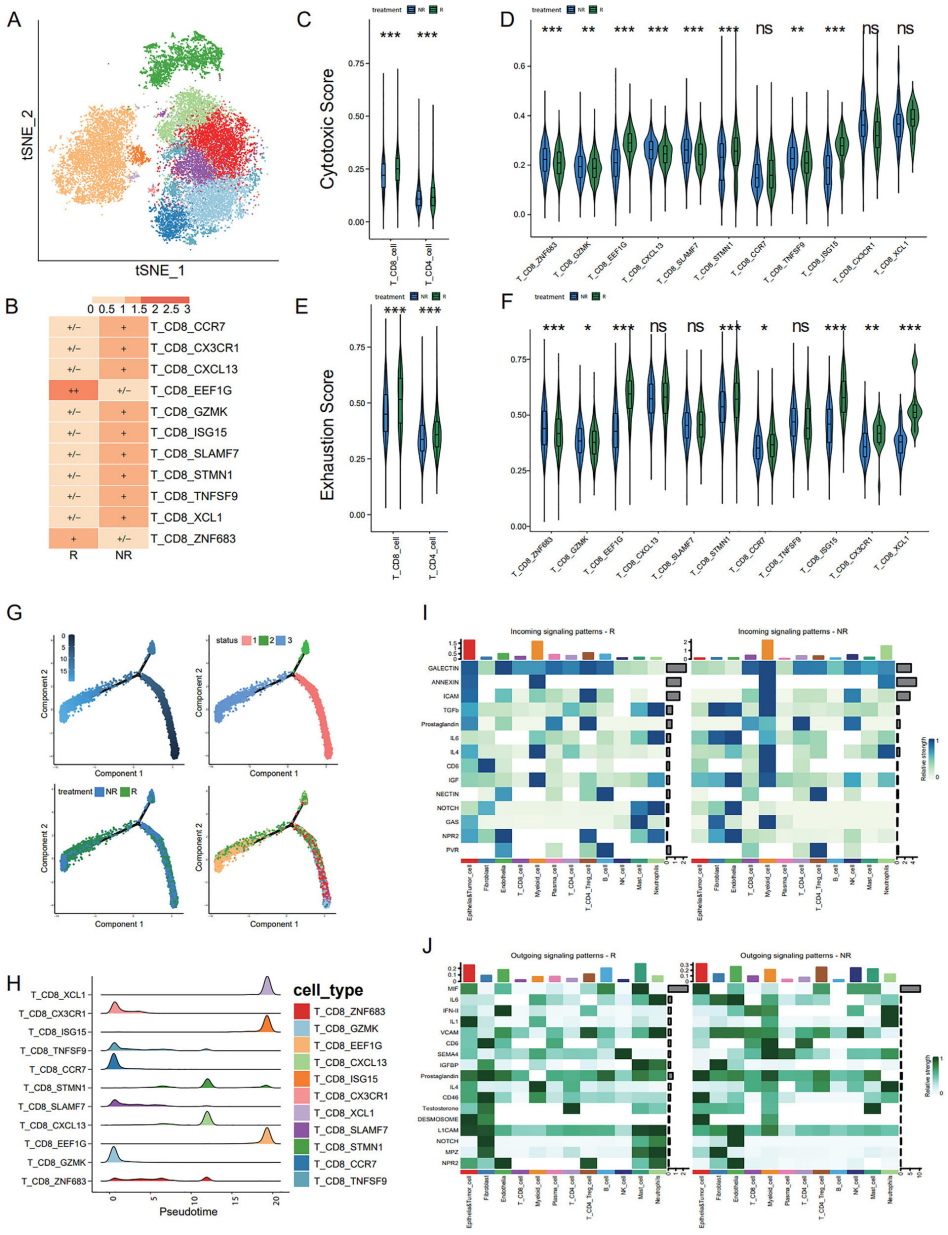
Functional profiling of the CD8^+^ T cell compartment and T–tumor interactions in responders (R) and non‐responders (NR). (A) t‐SNE visualization of CD8^+^ T‐cell subsets identified in the integrated single‐cell dataset. (B) Descriptive semi‐quantitative summary of CD8^+^ T cell subset enrichment in R and NR tumors. Columns indicate clinical response groups and rows indicate annotated CD8^+^ T cell subsets. Symbols summarize the relative enrichment tendency of each subset, derived from the direction and relative magnitude of the Ro/e analysis: ±, weak/intermediate enrichment; +, moderate enrichment; ++, strong enrichment. This panel is intended as a compact overview of response‐associated subset distribution patterns. (C,D) Cytotoxic score comparison between R and NR groups at the overall T_CD8_cell / T_CD4_cell level (C) and across individual CD8^+^ T‐cell subsets (D). (E,F) Exhaustion score comparison between R and NR groups at the overall T_CD8_cell / T_CD4_cell level (E) and across individual CD8+ T‐cell subsets. (G) Monocle2 pseudotime trajectory analysis of CD8^+^ T‐cell subsets, shown as inferred state‐transition maps colored by pseudotime, trajectory state, treatment group, and cell subtype, respectively. (H) Density distribution of annotated CD8^+^ T‐cell subsets along the inferred pseudotime trajectory. (I) Incoming signaling heatmap. Cell types are aligned across R and NR groups. Color intensity indicates the total incoming signaling strength, calculated as the sum of significant ligand–receptor interactions weighted by ligand/receptor expression. “Stronger” signaling refers to pathways with higher aggregated communication probability in R compared to NR tumors. (J) Outgoing signaling heatmap. Cell types are aligned across R and NR groups. Color intensity indicates outgoing signaling strength. Note: Only ligand–receptor interactions with *p* < 0.05 from permutation tests were included. Heatmaps are intended to illustrate qualitative trends in signaling strength, not to provide statistically tested quantitative comparisons.

We next focused on the CD8^+^ T cell compartment and found that its internal composition differed between responder and non‐responder tumors (Figure 4A,B). To provide a compact overview of these response‐associated differences, we generated a descriptive semi‐quantitative summary of CD8^+^ T cell subset enrichment based on the direction and relative magnitude of the Ro/e analysis (Figure 4B). This overview indicated that most CD8^+^ subsets, including T_CD8_CCR7, T_CD8_CX3CR1, T_CD8_CXCL13, T_CD8_GZMK, T_CD8_ISG15, T_CD8_SLAMF7, T_CD8_STMN1, T_CD8_TNFSF9, and T_CD8_XCL1, were relatively enriched in the non‐responder group, whereas T_CD8_EEF1G showed the strongest enrichment in the responder group and T_CD8_ZNF683 displayed a weaker responder‐skewed pattern (Figure 4B). These findings suggest that response‐associated remodeling within the CD8^+^ T cell compartment occurs primarily at the subset level, which was further supported by differences in cytotoxicity and exhaustion scores across individual CD8^+^ T cell states. We then compared functional scores across these CD8^+^ subsets. We then compared functional scores across these CD8^+^ subsets.

Both CD8^+^ and CD4^+^ T cells from R tumors displayed significantly higher overall cytotoxic scores, suggesting enhanced effector capacity. Within these lineages, CD8_PLAAT4, CD8_ISG15, CD4_EEF1G, and NK subsets (FCGR3A^+^ and AREG^+^) showed markedly increased cytotoxic activity in R samples, whereas CD8_CCL5, CD4_Treg, CD4_CCR7, CD4_IL7R, CD8_CXCL13, and CD4_CXCL13 subsets exhibited relatively diminished cytotoxic potential (Figure 4C,D). Conversely, exhaustion scores were generally elevated in R samples (Figure 4E,F), consistent with higher T cell activation and turnover following ICIs therapy. Similar to the cytotoxic pattern, CD8_PLAAT4, CD4_EEF1G, NK_FCGR3A, NK_AREG, and CD8_ISG15 populations displayed lower exhaustion, whereas CD8_CCL5 and CD4_Treg remained comparatively exhausted, highlighting functional heterogeneity within R tumors. To further resolve the dynamic organization of the CD8^+^ T cell compartment, we performed pseudotime trajectory analysis using Monocle2 (Figure 4G,H). This analysis reconstructed the inferred state‐transition landscape of CD8^+^ T cells and showed that distinct CD8^+^ T cell subsets occupied different positions along the trajectory. Notably, the responder‐associated subsets were preferentially distributed toward the later portion of the inferred pseudotime, whereas other subsets were concentrated in earlier or intermediate states. Consistent with the functional scoring results, subsets such as T_CD8_EEF1G, T_CD8_ISG15, T_CD8_XCL1, and T_CD8_STMN1 were more enriched in the later trajectory segments, supporting response‐associated remodeling at the level of CD8^+^ T cell states rather than total CD8^+^ T cell abundance alone. To define response‐associated intercellular crosstalk, we compared matched incoming and outgoing signaling programs between responder and non‐responder groups using the same cell‐type ordering across heatmaps (Figure 4I,J and Table S5D,E). In the responder group, CD8^+^ T cells and NK cells displayed higher incoming signaling through NPR2, PVR, GALECTIN, and NECTIN pathways, while Prostaglandin signaling was reduced relative to non‐responder samples. The total incoming signaling strength reflects the aggregated communication probability from all significant ligand‐receptor pairs within a pathway, weighted by ligand and receptor expression levels. The cell type order and color scheme are now consistent between R and NR groups, facilitating direct comparison. Heatmaps are presented in Figure 4I. In parallel, epithelial/tumor cells in the responder group displayed stronger CD6‐ and NOTCH‐related incoming signaling. Outgoing signaling analysis revealed a similar pattern, with stronger NPR2, Prostaglandin, and IFN‐II signaling from CD8^+^ T cells, and stronger NOTCH, L1CAM, and CD6 signaling from epithelial/tumor cells in the responder group. These findings support response‐associated remodeling of tumor‐immune communication patterns in NSCLC (Figure 4J and Figure S6A,B). Together, these findings define a comprehensive immune‐cell atlas of ICI‐treated NSCLC and establish a foundation for downstream analyses of activation states, ligand–receptor interactions, and spatial immune crosstalk.

### 3.5 GALNT7 Silencing Reduces Proliferation and Induces Apoptosis in NSCLC Cells

**FIGURE 5:**
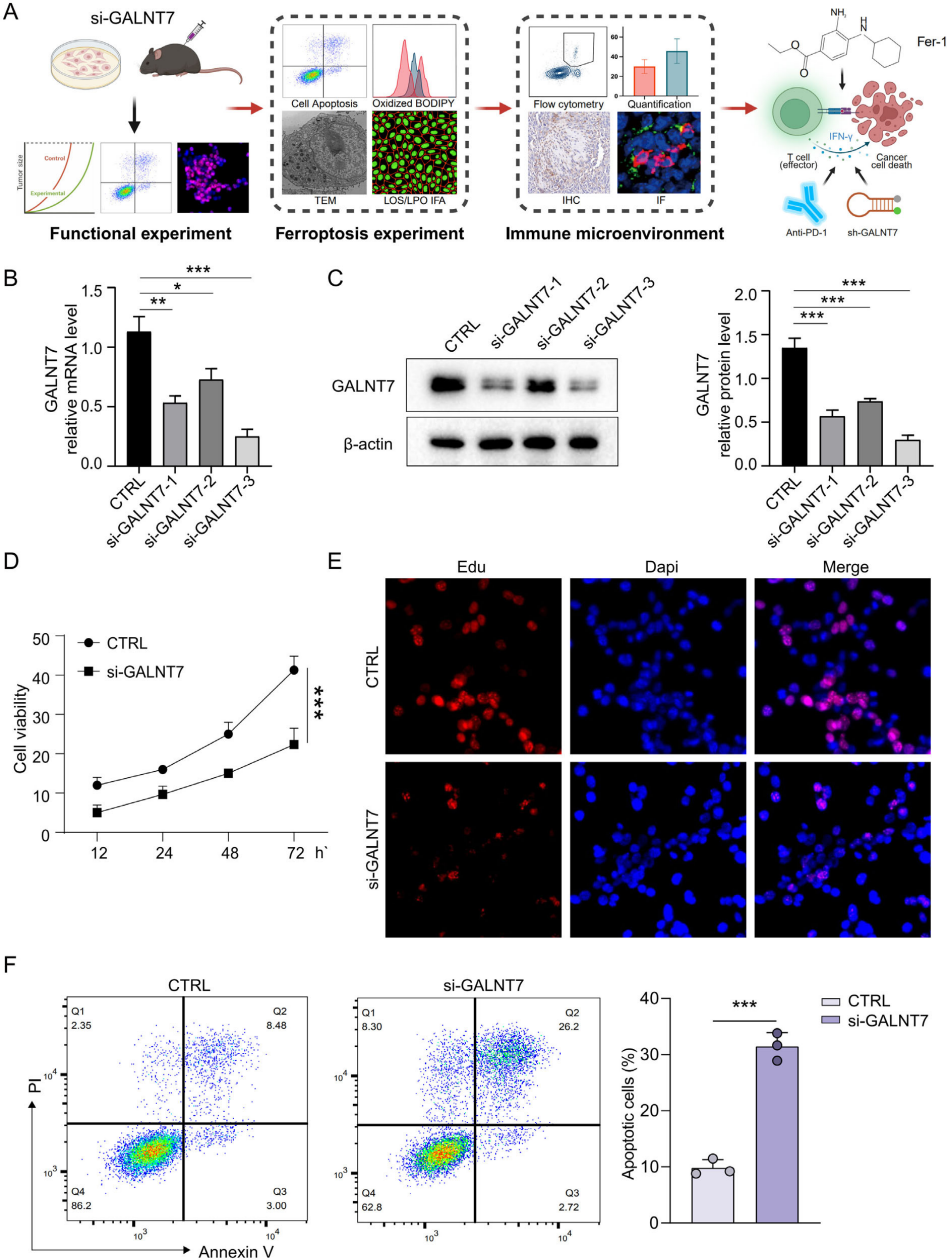
GALNT7 knockdown suppresses NSCLC cell proliferation and induces apoptosis. (A) Schematic workflow of GALNT7 functional assays, including ferroptosis, apoptosis, and immune microenvironment analyses. (B) qRT‐PCR showing reduced GALNT7 mRNA expression after transfection with three independent siRNAs compared with control. (C) Immunoblot analysis confirming decreased GALNT7 protein levels in the same setting, β‐actin served as loading control. The right panel shows quantification of GALNT7 protein levels in the corresponding setting. (D) Cell viability assay showing reduced proliferation following siGALNT7‐1 transfection. (E) EdU incorporation assay showing decreased DNA synthesis in siGALNT7‐1‐treated cells. (F) Annexin V/PI flow‐cytometric analysis showing increased apoptotic fractions after siGALNT7‐1‐mediated GALNT7 knockdown.

To determine the functional role of GALNT7 in NSCLC, we performed a series of in vitro assays following siRNA‐mediated knockdown (Figure 5A). Efficient depletion of GALNT7 was confirmed by qRT‐PCR and Western blotting, which showed a marked reduction (>70%) in both mRNA and protein levels across three independent siRNAs compared with control cells (*p* < 0.01; Figure 5B,C).

Using siGALNT7‐1 for the downstream functional assays, GALNT7 silencing led to a significant decrease in cell viability over a 72‐hour period, as measured by proliferation assays (*p* < 0.001; Figure 5D). Consistently, EdU incorporation assays demonstrated markedly reduced DNA synthesis in si‐GALNT7‐treated cells, indicating suppressed proliferative capacity (Figure 5E). Flow cytometric analysis of Annexin V/PI staining revealed a substantial increase in apoptotic cell fractions following siGALNT7‐1‐mediated GALNT7 knockdown (Figure 5F).

To ensure that this phenotype was not cell‐line specific, we repeated the same experiments in Calu‐3 cells [31], which yielded highly consistent results. GALNT7 knockdown reduced proliferation and promoted apoptosis to a comparable extent (Figure S7A–E).

Together, these findings demonstrate that GALNT7 is essential for maintaining NSCLC cell growth and survival, and that its suppression triggers apoptosis, highlighting GALNT7 as a potential therapeutic vulnerability in lung adenocarcinoma.

### 3.6 GALNT7 Depletion Activates Ferroptosis Signaling in NSCLC Cells

**FIGURE 6:**
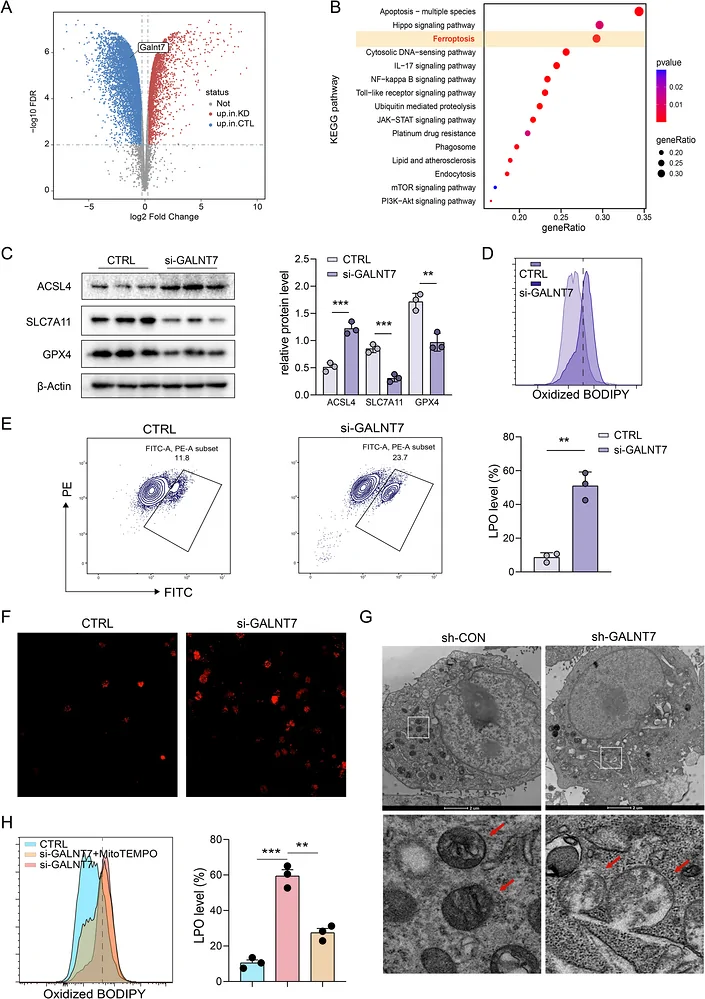
GALNT7 knockdown activates ferroptosis signaling in NSCLC cells. (A) Volcano plot showing DEGs following GALNT7 silencing. (B) KEGG pathway enrichment analysis showing significant enrichment of ferroptosis and lipid metabolism‐related pathways. (C) Immunoblot analysis of ferroptosis‐related proteins (ACSL4, SLC7A11, and GPX4) in control and si‐GALNT7 cells. β‐actin was used as a loading control. The right panel shows quantification of ACSL4, SLC7A11, and GPX4 protein levels. (D) Flow‐cytometric histograms of oxidized BODIPY fluorescence in control and siGALNT7 cells. (E) Flow‐cytometric measurement and quantification of lipid‐peroxidation (LPO) levels in control and siGALNT7 cells. (F) Fluorescence microscopy showing increased oxidized BODIPY signal in siGALNT7 cells relative to controls. (G) TEM images showing representative mitochondrial ultrastructural changes after GALNT7 silencing. The lower panels show enlarged views of the boxed regions; arrowheads indicate mitochondria with shrinkage, increased membrane density, and disrupted or reduced cristae. (H) Rescue experiment with MitoTEMPO showing attenuation of lipid peroxidation in GALNT7‐depleted cells, with corresponding quantification.

As a member of the acetylgalactosaminyltransferase family, GALNT7 plays a critical role in regulating mucin‐type O‐glycosylation [32], which influences tumor cell behavior by modulating protein stability and signaling at the cell surface [33]. To explore pathways associated with GALNT7 depletion, we performed bulk transcriptomic profiling of shGALNT7‐LLC and shNT‐LLC mouse tumors (Figure 6A and Table S3A). Differential gene expression analysis revealed a strong enrichment of ferroptosis‐associated genes, alongside pathways related to lipid metabolism, oxidative stress, and apoptotic signaling (Figure 6B and Table S3B–F). This suggests that, GALNT7 depletion might sensitize cells to ferroptosis, which is known to be tightly linked with lipid peroxidation and oxidative stress. Therefore, we hypothesize that the observed increase in tumor cell death due to GALNT7 loss could, in part, be attributed to ferroptosis activation.

To confirm activation of ferroptosis signaling, we further assessed the expression of core ferroptosis‐associated proteins [34]. ACSL4 promotes the incorporation of long‐PUFA acyl chains into phospholipids, increasing the pool of peroxidizable substrates and thereby sensitizing cells to ferroptosis. Consistent with the transcriptional changes, immunoblotting confirmed up‐regulation of the ferroptosis activator ACSL4, accompanied by significant down‐regulation of GPX4 and SLC7A11, two canonical ferroptosis suppressors (Figure 6C). We next analyzed known critical events in ferroptosis execution, such as mitochondrial morphology and lipid oxidation. Oxidized BODIPY‐C11 flow cytometry which quantifies membrane lipid peroxidation during ferroptosis was used [35, 36]. GALNT7 knockdown resulted in a pronounced rightward shift in oxidized BODIPY fluorescence, reflecting elevated lipid ROS accumulation (Figure 6D,E). Fluorescence microscopy further confirmed enhanced lipid peroxidation puncta in si‐GALNT7 cells relative to controls (Figure 6F). Moreover, TEM revealed representative ultrastructural changes consistent with ferroptosis, including shrunken mitochondria with condensed membranes density and disrupted or reduced cristae morphology (Figure 6G). In the enlarged panels, arrow annotations indicate the mitochondria showing these damage‐associated features. Similar ultrastructural alterations were also observed in Calu‐3 cells (Figure S8A–F).

This result is consistent with the findings previously observed in H460 cells following ferroptosis induction via ionizing radiation [37]. Notably, treatment with the mitochondria‐targeted antioxidant MitoTEMPO markedly rescued lipid peroxidation induced by GALNT7 silencing (Figure 6H), indicating that mitochondrial oxidative stress mediates this phenotype.

Notably, a similar ferroptotic response was observed in Calu‐3 cells following GALNT7 silencing, confirming that this mechanism is not cell‐line specific (Figure S8E).

Together, these findings establish that GALNT7 loss triggers ferroptosis in NSCLC cells, mediated through dysregulated lipid metabolism and mitochondrial ROS accumulation.

### 3.7 Ferroptosis Induction Enhances CD8^+^ T Cell Activation and Suppresses Tumor Growth In Vivo

**FIGURE 7:**
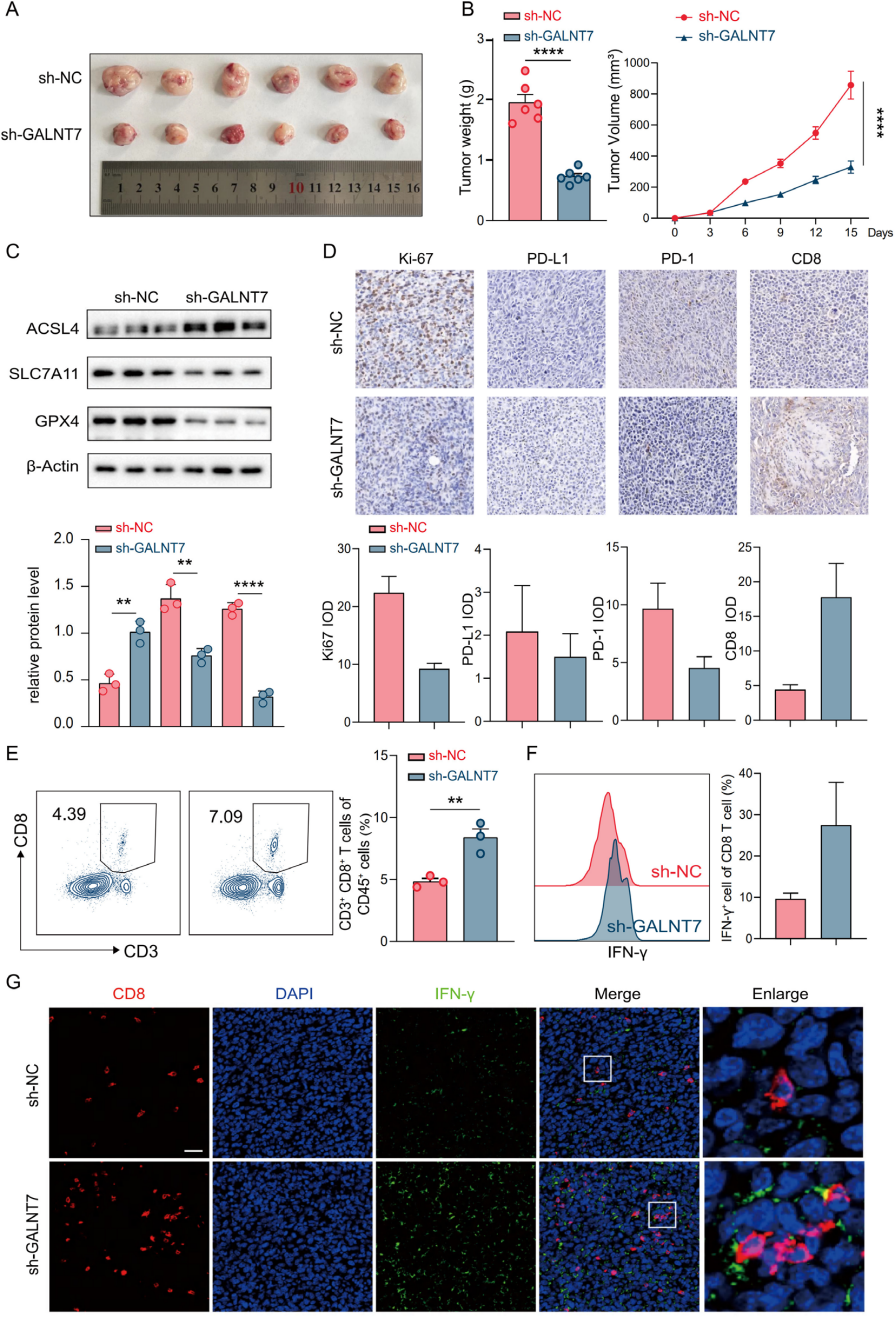
GALNT7 depletion suppresses tumor growth and enhances CD8^+^ T cell responses in vivo. (A) Representative images of syngeneic LLC tumors derived from sh‐GALNT7 and sh‐NC NSCLC cells. (B) Quantification of tumor weight (left) and growth curves (right) showing reduced tumor burden in the sh‐GALNT7 group. (C) Western blot analysis of ACSL4, SLC7A11, and GPX4 in tumor tissues from sh‐NC and sh‐GALNT7 tumors. The lower/right panel shows group‐labeled densitometric quantification of the corresponding protein levels. (D) Immunohistochemical staining of Ki‐67, PD‐L1, PD‐1, and CD8 in sh‐NC and sh‐GALNT7 tumors. The lower panels show quantitative analysis of Ki‐67, PD‐L1, PD‐1, and CD8 staining with statistical comparison between groups. (E) Flow cytometric analysis and quantification of tumor‐infiltrating CD3^+^CD8^+^ T cells in sh‐NC and sh‐GALNT7 tumors. (F) Flow‐cytometric analysis of IFN‐γ–related immune activation in tumor‐infiltrating lymphocytes from sh‐NC and sh‐GALNT7 tumors. The corresponding quantitative analysis and statistical comparison are shown in the associated panels. (G) Immunofluorescence staining showing colocalization of CD8 (red) and IFN‐γ (green) signals, indicating enhanced cytotoxic T cell activation in sh‐GALNT7 tumors.

Having established a ferroptosis program upon GALNT7 loss in vitro, we validated this mechanistic axis in vivo. We established a syngeneic subcutaneous LLC tumor model in C57BL/6J mice using sh‐GALNT7–transduced NSCLC cells. Compared with control (sh‐NC) tumors, GALNT7 depletion markedly inhibited tumor growth, as evidenced by reduced tumor volume and weight (Figure 7A,B). Western blot analysis of tumor tissues further showed increased ACSL4 expression together with decreased SLC7A11 and GPX4 levels in the sh‐GALNT7 group, and the corresponding densitometric quantification is shown in Figure 7C. Previous studies have shown that ferroptosis can modulate the TIME by releasing damage‐associated molecules that enhance antigen presentation and T cell recruitment [17, 38]. Given this link between ferroptosis and immune activation, we examined how GALNT7‐induced ferroptosis affected immune infiltration in vivo. Immunohistochemical quantification demonstrated significantly decreased Ki‐67, PD‐L1, and PD‐1 staining, together with significantly increased CD8^+^ cell infiltration, in sh‐GALNT7 tumors relative to controls (Figure 7D). Flow‐cytometric quantification further showed a higher proportion of tumor‐infiltrating CD3^+^CD8^+^ T cells in the sh‐GALNT7 group (Figure 7E). Consistently, IFN‐γ‐related immune activation was increased in the sh‐GALNT7 group, as supported by the corresponding quantitative analysis and statistical comparison (Figure 7F,G).

Collectively, these findings indicate that GALNT7 loss not only suppresses tumor growth but also reshapes the immune microenvironment by triggering tumor‐cell ferroptosis, which in turn enhances CD8^+^ T cell activation and anti‐tumor immunity.

### 3.8 GALNT7 Loss Synergizes with PD‐1 Blockade to Enhance Anti‐Tumor Immunity via Ferroptosis Activation

**FIGURE 8:**
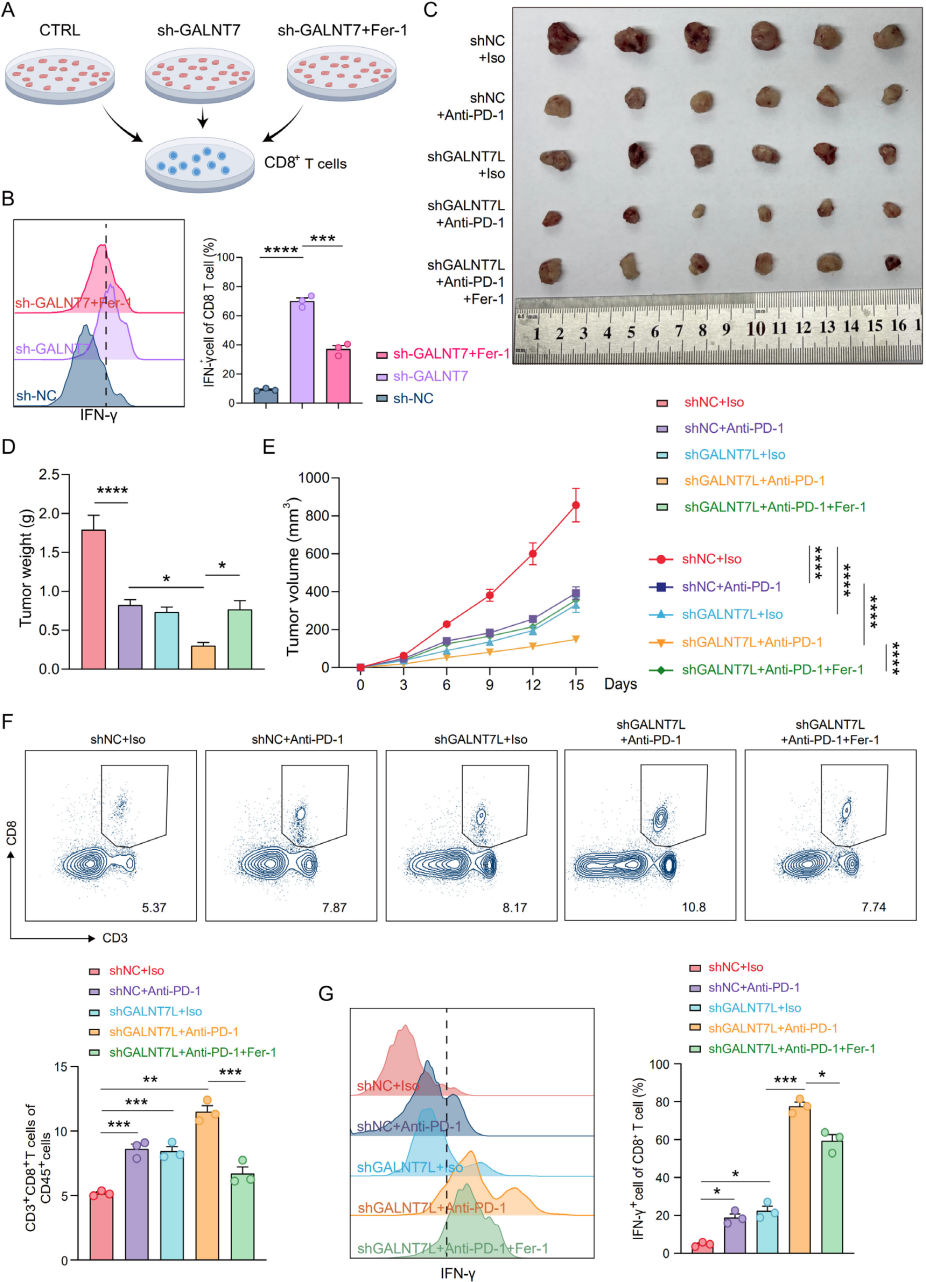
GALNT7 knockdown synergizes with PD‐1 blockade to enhance anti‐tumor immunity through ferroptosis activation. (A) Schematic of the co‐culture and in vivo treatment design combining GALNT7 knockdown, PD‐1 blockade, and ferroptosis inhibition (Fer‐1). (B) Flow‐cytometric analysis of IFN‐γ production in CD8^+^ T cells isolated from tumor‐bearing mice, showing increased cytokine release after GALNT7 silencing and attenuation by Fer‐1. The right panel shows quantification of IFN‐γ^+^ CD8^+^ T cell frequency (%). (C) Representative images of excised tumors from each treatment group (shNC + Iso, shNC + Anti‐PD‐1, shGALNT7L + Iso, shGALNT7L + Anti‐PD‐1, shGALNT7L + Anti‐PD‐1 + Fer‐1). (D,E) Quantification of tumor weight (left) and growth curves of tumor volume (right) demonstrating maximal suppression by GALNT7 loss combined with PD‐1 blockade, with partial rescue by Fer‐1 treatment. (F) Flow cytometry plots and bar graph quantification of tumor‐infiltrating CD3^+^CD8^+^ T cells among CD45^+^ immune cells, indicating enhanced T cell infiltration in the shGALNT7 + Anti‐PD‐1 group and reversal with Fer‐1. (G) Flow‐cytometric histograms of IFN‐γ expression and quantification of IFN‐γ levels in tumor‐infiltrating CD8^+^ T cells, confirming ferroptosis‐dependent immune activation. Data are presented as mean±SEM. P values were calculated using two‐tailed Student's t test or one‐way ANOVA with Tukey's correction.

Given that GALNT7 depletion promoted ferroptosis and enhanced anti‐tumor immune activity, we next examined whether its loss could potentiate ICB. To this end, we first conducted in vitro assays using 3 groups: sh‐GALNT7, sh‐GALNT7 + Ferrostatin‐1 (Fer‐1), and control (shNC) tumors. We assessed IFN‐γ production by co‐culturing CD8^+^ T cells isolated from these tumor‐bearing mice (Figure 8A). Co‐culture assays demonstrated that CD8^+^ T cells isolated from sh‐GALNT7 tumors displayed significantly increased IFN‐γ production, indicating enhanced cytotoxic activation. Remarkably, this effect was largely reversed upon treatment with Fer‐1, suggesting that ferroptosis contributes to the activation of effector T cells (Figure 8B).

In vivo, we established a syngeneic subcutaneous LLC tumor model with five treatment groups: shNC+Iso, shNC+Anti‐PD‐1, shGALNT7+Iso, shGALNT7+Anti‐PD‐1, and shGALNT7+Anti‐PD‐1+Fer‐1 (Figure 8C). GALNT7 silencing or PD‐1 blockade alone reduced tumor burden, while their combination produced the strongest anti‐tumor effect, reflected by markedly decreased tumor volume and weight (Figure 8C–E). Conversely, co‐administration of Fer‐1 partially abrogated the tumor‐suppressive effect of the combined treatment (Figure 8C–E). Flow cytometric analysis revealed a substantial increase in tumor‐infiltrating CD3^+^CD8^+^ T cells in the sh‐GALNT7+anti‐PD‐1 group, which was again diminished by Fer‐1 treatment (Figure 8F). Likewise, IFN‐γ production in CD8^+^ T cells was highest in the combined therapy group and decreased following ferroptosis inhibition (Figure 8G).

Together, these results indicate that GALNT7 depletion synergizes with PD‐1 blockade to potentiate anti‐tumor immunity, and that ferroptosis activation is a key mediator of this enhanced therapeutic response. This underscores the potential of combining ferroptosis modulation with ICB to overcome resistance and improve treatment outcomes.

In conclusion, our study highlights the pivotal role of GALNT7 in regulating ferroptosis and tumor immunity. We demonstrate that GALNT7 depletion activates ferroptosis signaling, resulting in enhanced tumor cell death and remodeling of the immune microenvironment, particularly through increased CD8^+^ T cell infiltration and IFN‐γ production. Furthermore, our findings suggest that GALNT7 loss synergizes with PD‐1 blockade, potentiating anti‐tumor immunity via ferroptosis activation. Importantly, ferroptosis inhibition with Fer‐1 partially reversed the therapeutic effects of GALNT7 depletion, underscoring the critical role of ferroptosis in mediating immune responses. These results provide a metabolic framework where targeting GALNT7 and ferroptosis can offer new therapeutic strategies for overcoming ICB resistance in lung cancer.

## 4 Discussion

Our study reveals that ferroptotic vulnerability within tumor epithelial cells is a determinant of effective anti‐tumor immunity in NSCLC. Through integrated single‐cell, spatial, and bulk transcriptomics from patients treated with ICB, we demonstrate that responders exhibit enhanced lipid‐peroxidation programs and higher ferroptosis scores in malignant regions, accompanied by greater infiltration and cytotoxic activity of CD8^+^ T cells. GALNT7 emerged as a functional regulator: tumors with high GALNT7 expression displayed suppressed ferroptotic signatures and reduced immune engagement, whereas depletion of GALNT7 restored ferroptotic sensitivity and potentiated PD‐1 blockade efficacy that were blunted by pharmacologic inhibition of ferroptosis. Together, these data suggest that tumor‐cell ferroptosis acts as a lever for immune responsiveness, and that GALNT7 contributes to immune escape by restricting this form of regulated cell death.

Immunotherapy has fundamentally changed the treatment landscape of advanced NSCLC, yet durable benefit remains limited to a minority of patients. PD‐1/PD‐L1 monotherapy produces ORRs of ≈20%–45% depending on PD‐L1 expression (≈19% in previously treated, ≈27% with PD‐L1 ≥ 1%, ≈45% with PD‐L1 ≥ 50%) [39, 40], while chemo‐immunotherapy can increase ORR toward ≈40%–60% [41]; nonetheless, primary and acquired resistance remain common, with over half of initial responders relapsing during follow‐up [42]. Resistance arises from both intrinsic and extrinsic mechanisms: reduced antigenicity through MHC or B2M loss, aberrant oncogenic signaling that restricts lymphocyte infiltration, and suppressive microenvironmental circuits maintained by myeloid cells, Tregs, and dysfunctional T or NK cells [43, 44, 45, 46].

In keeping with prior studies of the TIME, our single‐cell analysis suggested that responsive tumors were associated with more favorable CD8^+^ T cell functional states, including higher cytotoxic activity, whereas the broad lineage‐level composition differences at the sample level were comparatively limited. These results are consistent with observations in several solid tumors that the states of pre‐existing effector T cells, rather than PD‐L1 expression alone, predict benefit from ICB treatment [47, 48]. Although minor differences in cell type proportions were observed between R and NR tumors, these differences are descriptive and should be interpreted with caution due to compositional dependencies and sample‐specific effects. The integrated single‐cell atlas primarily highlights qualitative trends rather than statistically significant quantitative differences in lineage composition. A distinctive feature of our dataset lies in the metabolic programs of epithelial cells. Tumor regions from responsive patients displayed coordinated enrichment of lipid‐metabolic and ferroptosis‐related pathways. However, resistant lesions showed stronger expression of genes associated with glycosylation, particularly GALNT7. This divergence suggests that ferroptotic competence may underlie an immune‐permissive state. Ferroptosis represents a specialized, iron‐dependent death program characterized by accumulation of lipid peroxides and oxidative damage to cellular membranes [49, 50, 51]. Because this process can be triggered by depletion of cystine, inhibition of GPX4, or increased ACSL4‐mediated lipid incorporation, it is highly sensitive to metabolic re‐wiring within tumor cells.

Accumulating evidence connects ferroptosis to anti‐tumor immunity [38, 52]. Activated CD8^+^ T cells can potentiate tumor ferroptosis through interferon‐γ driven repression of SLC7A11, amplifying lipid peroxidation and tumor clearance [53, 54]. Radiotherapy and certain chemotherapeutic agents also converge on this pathway, lowering SLC7A11 expression and thereby enhancing responsiveness to immunotherapy [37, 55]. Our data extend this paradigm by identifying GALNT7 as a tumor‐intrinsic brake on this process. We show that GALNT7 maintains a ferroptosis‐resistant state by stabilizing GPX4 and SLC7A11 expression, which in turn limits IFN‐γ‐STAT1‐driven oxidative stress and cytotoxic T cell infiltration. Our findings indicated that tumor cell glycosylation programs directly shape ferroptotic susceptibility and, consequently, the immune landscape.

Nonetheless, ferroptosis in cancer is not uniformly beneficial. On the one hand, ferroptotic tumor cells can emit DAMPs such as HMGB1 or ATP and expose calreticulin, events that promote dendritic‐cell maturation and antigen cross‐presentation [56, 57]. On the other hand, excessive lipid oxidation in immune or stromal subsets may have opposite effects. Ferroptotic neutrophils and MDSCs release prostaglandins and oxidized lipids that blunt CD8^+^ T cell function, and effector T cells themselves depend on GPX4 to resist ferroptotic stress [58, 59, 60]. Our integrated human and murine data indicate that, within NSCLC treated with PD‐1 blockade, the net effect of tumor‐cell ferroptosis is anti‐tumoral. However, the immunologic outcome of ferroptosis appears stage‐dependent—early events stimulate antigen presentation, whereas late or excessive ferroptosis favors immunosuppression. Thus the timing and extent of ferroptosis should be considered when designing therapeutic interventions.

Functionally, GALNT7 modulates ferroptosis through its role in protein O‐glycosylation [61]. GALNT family enzymes initiate mucin‐type O‐glycosylation, shaping protein stability and signaling at the plasma membrane [62]. GALNT7 has been implicated in proliferation and metastasis in thyroid carcinoma and prostate cancer [63, 64], but its connection to ferroptosis has not been described previously. Our experiments suggest that elevated GALNT7 disrupts ferroptotic sensitivity, possibly by modifying or stabilizing components of the xCT/GPX4 axis. Loss of GALNT7 re‐sensitized tumor cells to ferroptosis and improved response to PD‐1 therapy, pointing to a glycosylation interface that governs immune recognition. Identifying the direct glycoprotein substrates of GALNT7 will be essential for clarifying this mechanism.

Recent studies have shown that excessive lipid peroxidation within the TME can directly compromise T cell function. Oxidized lipid accumulation can impair T cell receptor signaling, while CD36‐mediated uptake of lipid peroxides drives T cell exhaustion; GPX4 is required to protect T cells from lipid‐ROS‐induced death [65, 66]. In our responder group, tumor‐cell ferroptosis correlated with heightened CD8 cytotoxicity rather than T cell depletion, suggesting that ferroptosis was largely confined to malignant cells and that antigen release and cytokine cues outweighed potential oxidative damage to immune cells. Overall, early ferroptosis restricted in tumor cells under IFN‐γ/STAT1 signaling, with ATP/HMGB1 release and calreticulin exposure, is immunogenic [67], whereas late ferroptosis or ferroptosis in T cells or myeloid populations with excess oxidized lipids/PGE2 is suppressive [68]. Therapeutic design should amplify the early immunogenic phase while avoiding immune‐cell ferroptosis.

From a translational standpoint, the combination of GALNT7 suppression with PD‐1 blockade enhanced anti‐tumor efficacy, whereas pharmacologic inhibition of ferroptosis partially reversed this effect. These data implicate ferroptosis as the mechanistic bridge connecting GALNT7 activity and immune responsiveness. Although clinically practical ferroptosis inducers are still under development, small molecules such as imidazole ketone erastin and cystinase have validated the feasibility of pharmacologic ferroptosis induction and demonstrated synergy with checkpoint blockade in preclinical models [69]. Our work thus provides rationale for assessing ferroptosis‐related biomarkers, including GALNT7 expression and ferroptosis signatures, as predictive tools in future trials combining ferroptosis modulators with immunotherapy. Because O‐glycosylation enzymes are druggable, targeting GALNT7 directly might also represent a strategy to reprogram resistant tumors toward ferroptotic vulnerability and immune activation [64].

Several limitations should be acknowledged. Mechanistically, we have not yet delineated how ferroptosis in tumor cells influences T cell recruitment or antigen presentation at the molecular level. The glycoprotein substrates through which GALNT7 affects ferroptotic pathways remain undefined, and our experimental models cannot fully reproduce the heterogeneity of human NSCLC. Although uniform QC filtering and Harmony‐based integration were applied, residual effects of pre‐analytical differences between in‐house and public datasets, including differences in mitochondrial RNA content, cannot be completely excluded. In addition, the integrated human cohort showed a certain degree of heterogeneity with respect to clinical stage, treatment setting, and data source. Moreover, spatial transcriptomic validation was based on only a single non‐responder case. Given the spot‐level resolution and the absence of formal spatial correlation testing, these spatial observations should be interpreted as illustrative and supportive rather than as statistically conclusive or broadly generalizable evidence. Therefore, the human transcriptomic data should be interpreted primarily as a resource for identifying resistance‐associated cellular states rather than as a fully balanced clinical comparison cohort. In addition, because fresh tumor dissociation was performed enzymatically at 37°C, we cannot fully exclude dissociation‐induced transcriptional perturbations, particularly activation‐ or stress‐associated signatures in immune cells such as T cells.

Expanding these findings into larger clinical cohorts and mechanistic studies using conditional alleles or single‐cell multi‐omics will be crucial to validate causality and clinical utility.

Overall, the present study suggests that ferroptosis is an actionable component of the immune response in NSCLC and positions GALNT7 as both a prognostic marker and a potential therapeutic target. Integrating ferroptosis modulation into immunotherapy may provide a means to convert immune‐cold tumors into responsive ones, thereby broadening the benefit of checkpoint blockade.

## Author Contributions

**G.Y.J**.: conceptualization and supervision. **J.D.G**., **Q.Z**., **D.L**., **R.C.N**., **M.L.Y**., **X.L.T**.: results analysis and visualization. **D.L**., **X.C.Z**., **M.L.Y**.: guidance on experiments. **R.J.X**., **J.H.X**.: experimental operations. **W.J.M**., **W.X.L**., **D.L**.: clinical samples collection; **J.D.G**., **Q.Z**., **D.L**.: writing – original draft with all authors contributing to providing feedback.

## Ethics Statement

This study was approved by the Laboratory Animal Ethics Committee of West China Hospital, Sichuan University (Approval No. 20240923015), and all animal procedures were carried out in accordance with institutional animal welfare regulations and the predefined humane endpoint criteria described in the Methods section. Patients in this study were included with informed consent and institutional ethical approval (Protocol No. 2018.270).

## Conflicts of Interest

The authors declare no conflicts of interest.

## Supporting information




**Supporting File 1**: advs76082‐sup‐0001‐SuppMat.docx.

**Supporting File 2**: advs76082‐sup‐0002‐FigureS1‐S8.zip.

**Supporting File 3**: advs76082‐sup‐0003‐TableS1‐S6.zip.

## Data Availability

The in‐house single‐cell and spatial transcriptomic RNA sequencing and data used to support the findings of this study are available from the corresponding author (Guiyi Ji, guiyi01@wchscu.cn) upon reasonable request. The public single‐cell RNA‐seq data used in this study are available from Gene Expression Omnibus, under accession number GSE207422 (https://www.ncbi.nlm.nih.gov/geo/query/acc.cgi?acc=GSE207422).
