# Supplementary material for: Single‐Cell Reveal GALNT7‐Dependent Ferroptosis Suppression as a Mechanism of Immunotherapy Resistance in Non‐Small Cell Lung Cancer
Source: Adv Sci (Weinh). 2026 Jun 19:e76082. Online ahead of print. doi: 10.1002/advs.76082 (PMC13336806; doi:10.1002/advs.76082)
Supplement: Supplementary file 1 — Supporting File 1: advs76082‐sup‐0001‐SuppMat.docx. [file ADVS-9999-e76082-s003.docx]

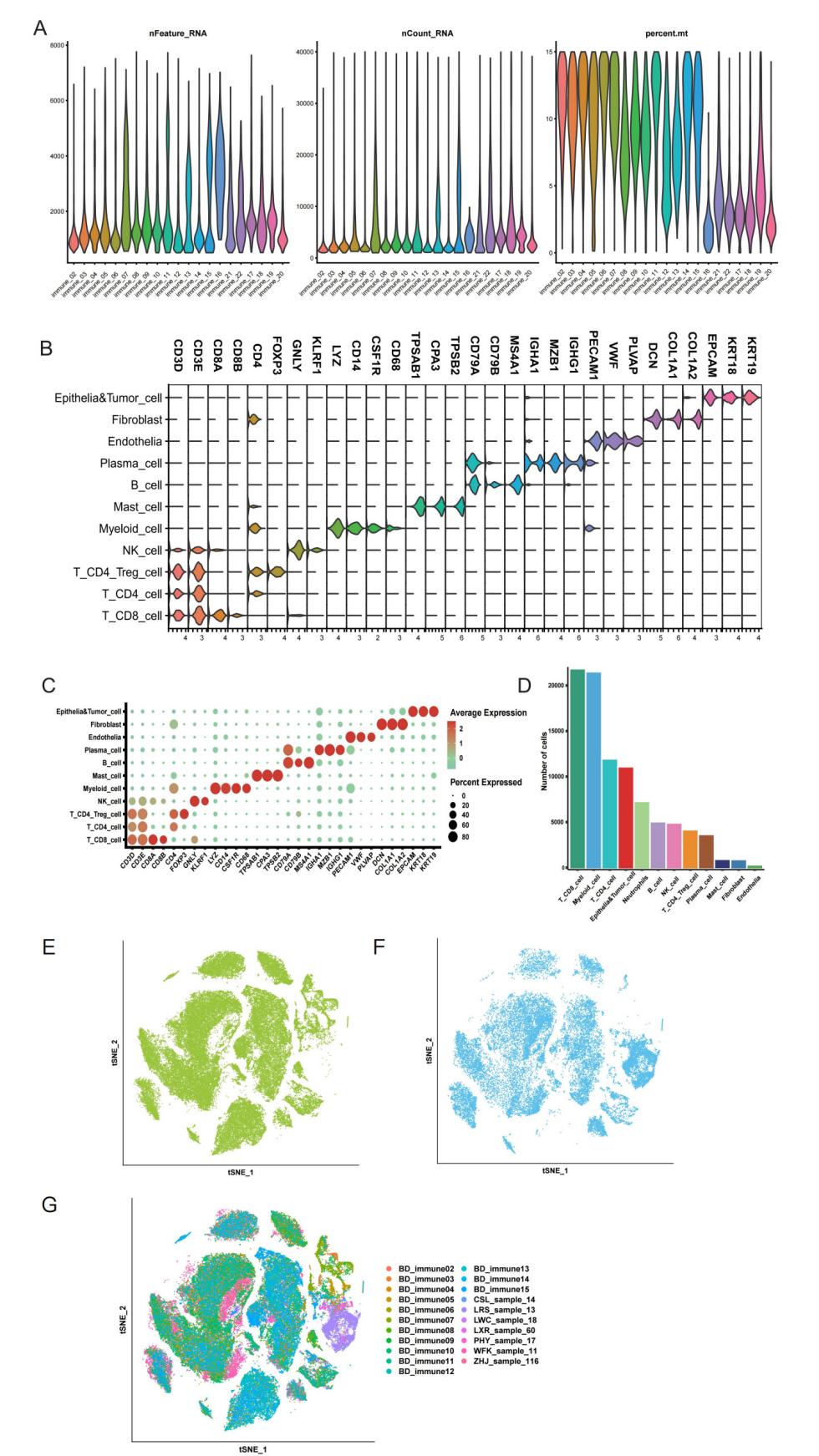


### Figure S1. Single-cell characterization of NSCLC tumor.

(A) Violin plots showing the distribution of detected genes, total UMI counts, and mitochondrial transcript percentages across annotated cell clusters.

(B) Violin plots depicting canonical marker expression across major cell lineages.

(C) Dot plot summarizing the average expression and frequency of lineage-defining markers across identified clusters.

(D) Bar chart quantifying the total number of cells per annotated cell type.

(E) tSNE plot of single-cell RNA-seq data from public cohorts, showing the distribution of responders (R) or non-responders (NR) cells. Colors indicate data source identity. Minimal batch effects are observed after integration.

(F) tSNE plot of single-cell RNA-seq data from in-house cohort, showing the distribution of R and NR cells. Colors indicate individual sample identity. Integration demonstrates minimal batch effects across samples.

(G) tSNE plot of all individual samples combined, showing the distribution of R and NR cells. Each color represents a unique patient sample. These plots confirm that batch effects are minimal after integration.

**
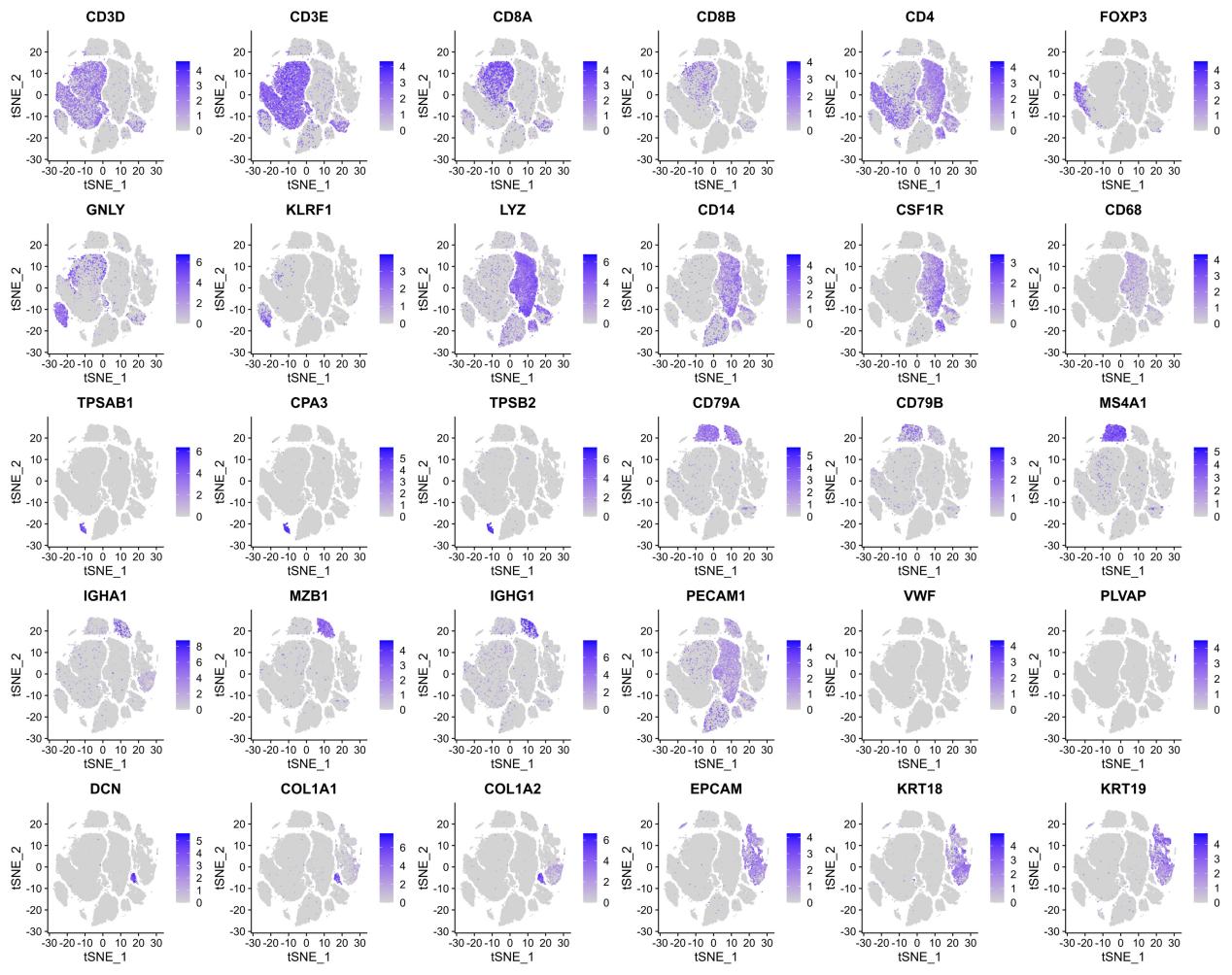
**

**Figure S2. Marker gene expression defining major cell lineages in NSCLC tumor samples.** t-SNE plots showing the expression patterns of canonical marker genes across all cells profiled in the dataset. Each dot represents a single cell, colored according to normalized expression level (purple gradient).

**
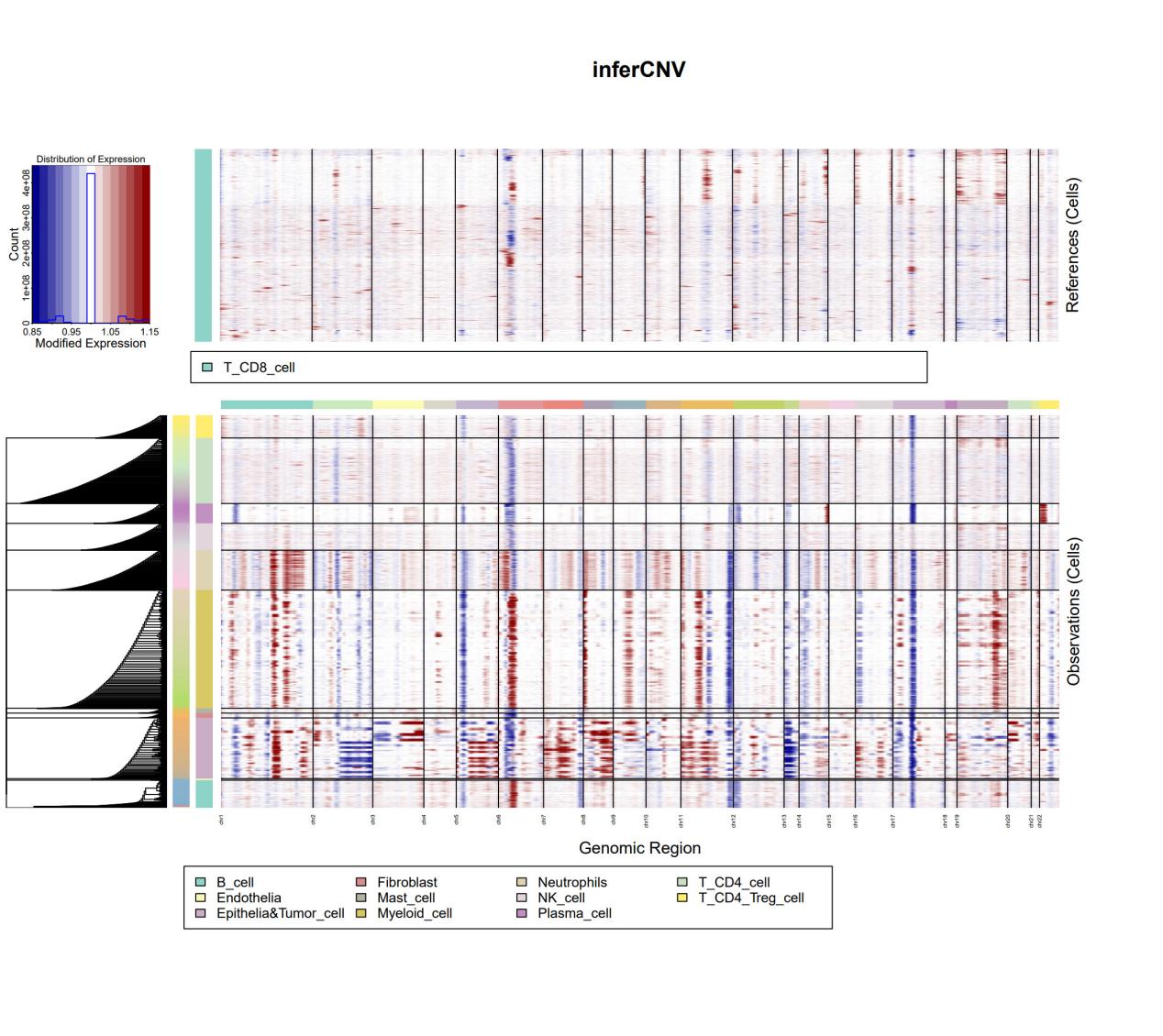
**

**Figure S3. Copy-number variation analysis confirming malignant cell identity.** InferCNV analysis was performed using CD8^+^ T cells as the reference population (top) and epithelial/tumor cells as the test population (bottom). Each column represents a genomic region ordered by chromosomal position, and each row corresponds to a single cell. Red indicates relative gains, blue indicates losses. Widespread CNV signals across multiple chromosomal regions support the malignant identity of epithelial/tumor clusters.

**
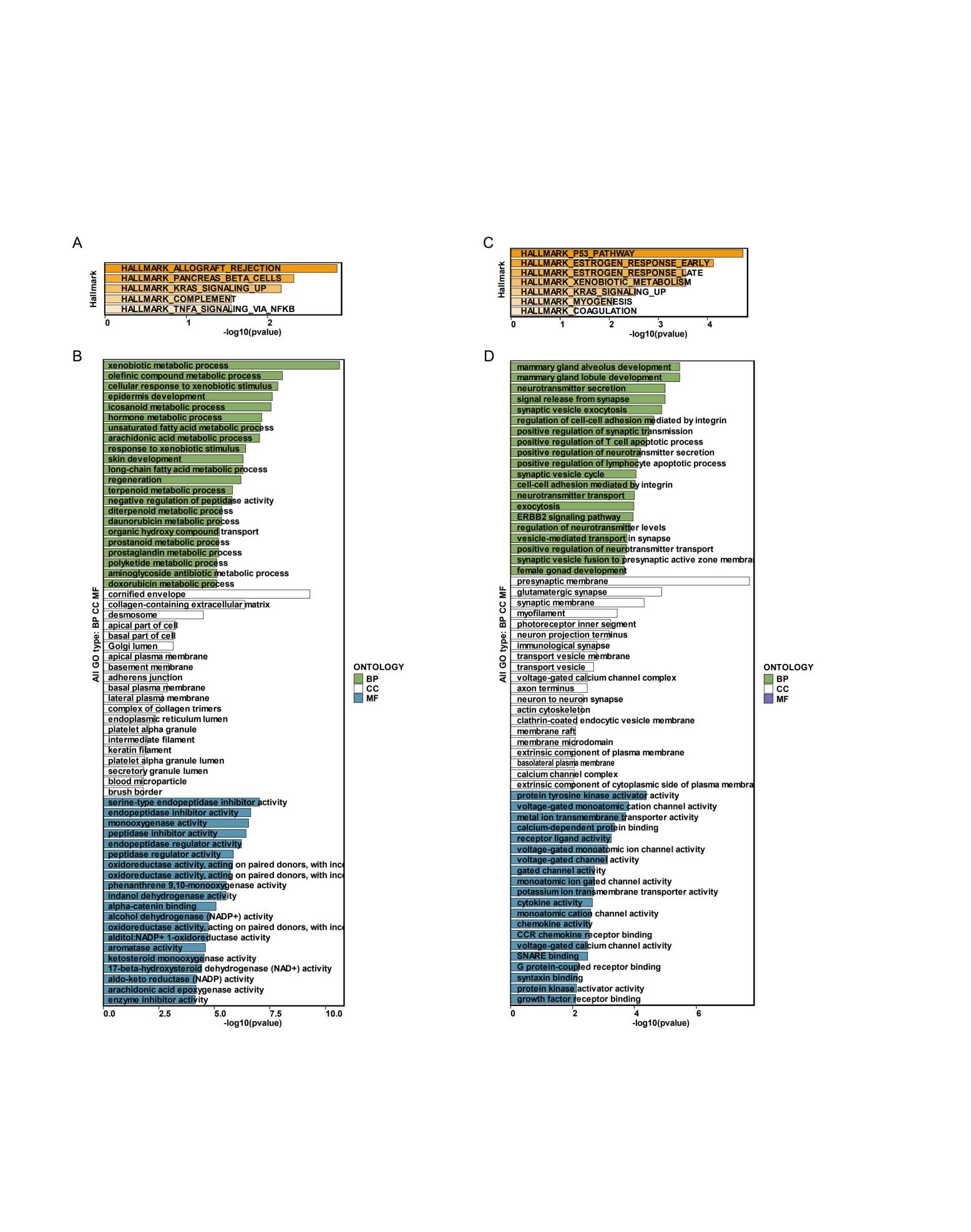
**

**Figure S4. Functional enrichment analysis of responder (R) and non-responder (NR) epithelial populations.**

(A, B) Corresponding enrichment analysis in R tumors showing increased oxidative phosphorylation, fatty acid metabolism, and ferroptosis-related processes.

(C, D) Hallmark and GO enrichment analyses of upregulated genes in NR tumor epithelial cells, highlighting activation of inflammatory and epithelial-mesenchymal transition (EMT) pathways.


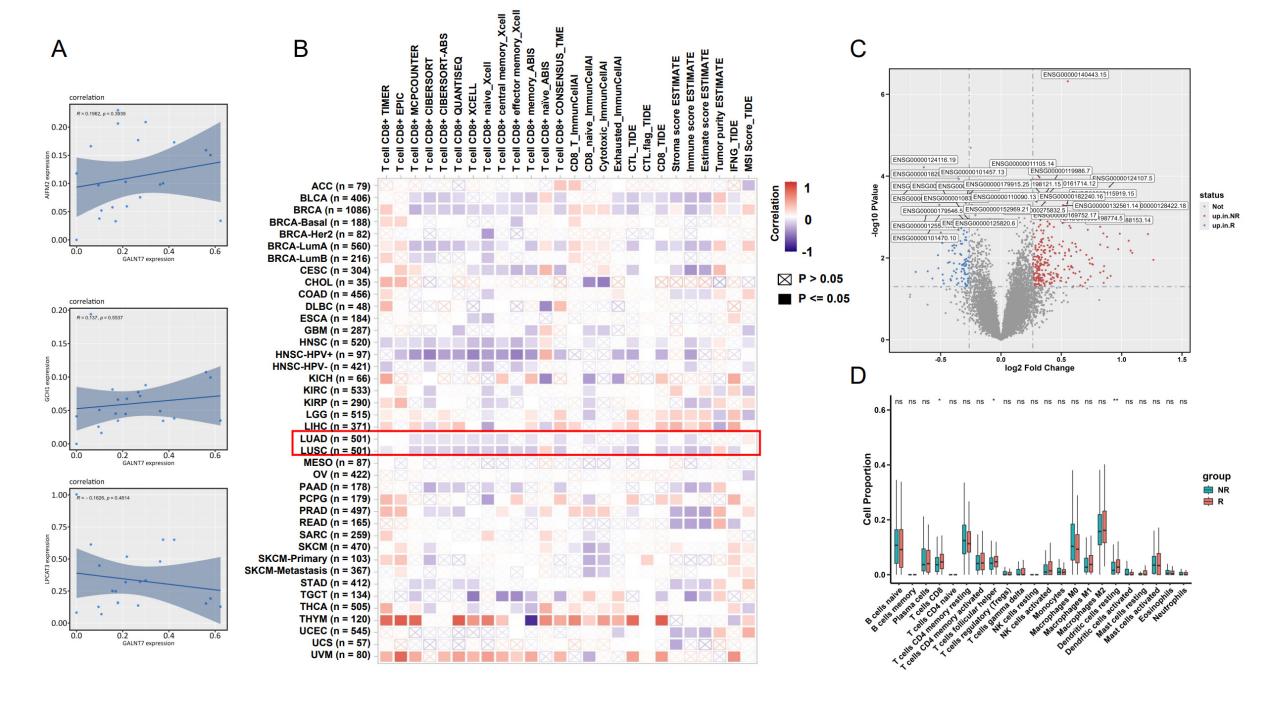


**Figure S5. Bulk-level validation of response-associated transcriptional and immune-compositional differences, together with supportive donor-level ferroptosis-related correlation analyses.**

(A) Donor-level scatterplots showing the relationships between GALNT7 expression and three representative ferroptosis-related genes across epithelial/tumor-cell samples, including the ferroptosis suppressors AIFM2/FSP1 and GCH1, and the ferroptosis driver LPCAT3. Each point represents one donor/sample, and Spearman correlation coefficients and P values are shown in the panels.

(B) Pan-cancer correlation analysis between GALNT7 expression and immune-cell infiltration across TCGA cancer types using multiple immune deconvolution methods.

(C) Differential gene-expression comparison between responder (R) and non-responder (NR) samples in the external immunotherapy-treated NSCLC bulk RNA cohort. Horizontal axis labels indicate individual genes, and the y-axis shows the log2 fold change between R and NR groups.

(D) Transcriptome deconvolution analysis showing inferred tissue-level cell proportions in responder and non-responder samples from the same cohort.


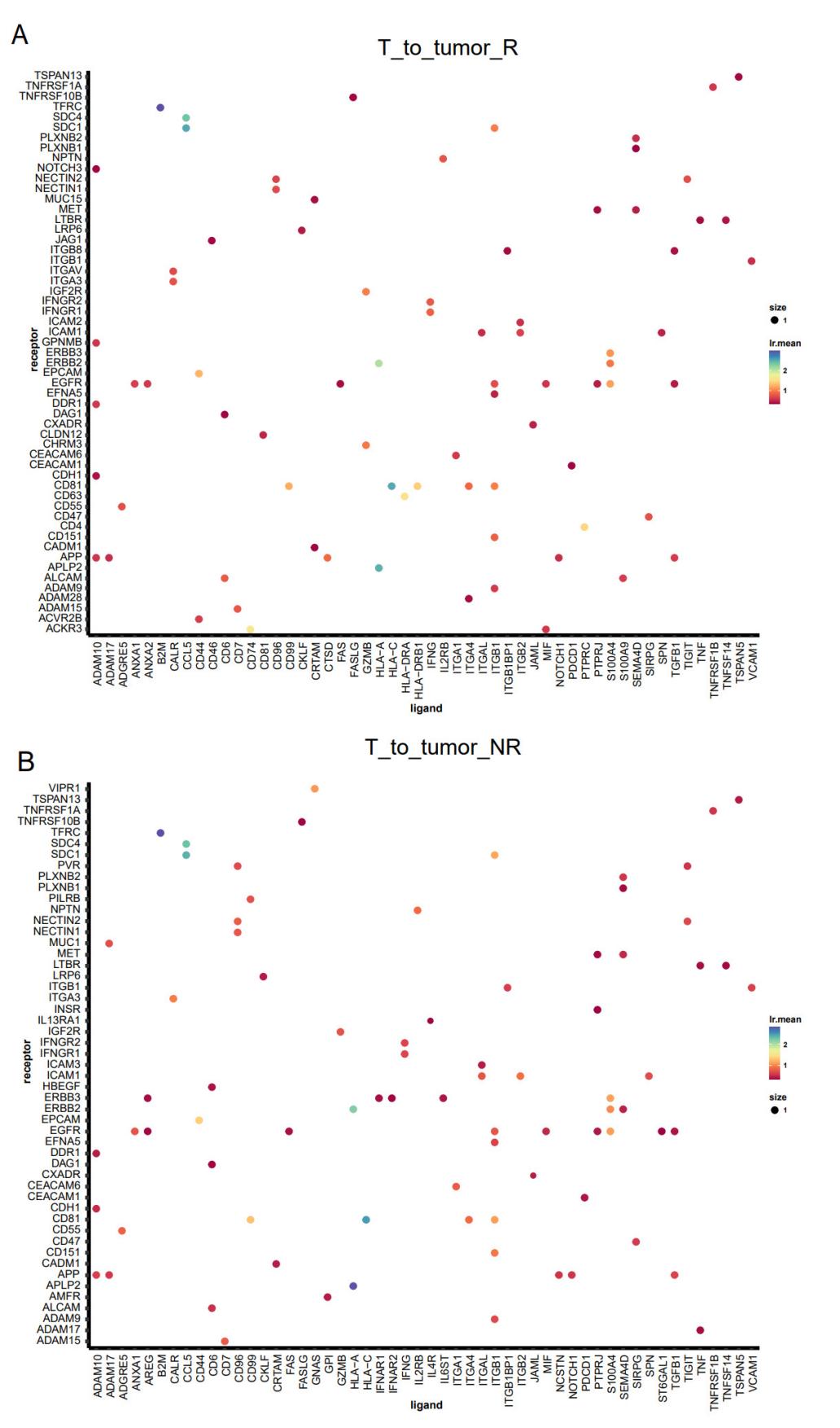


**Figure S6. Differential epithelial-centric signaling in responders (R) versus immune-skewed signaling in non-responders (NR).**
(A) R samples. Sphere plot indicates a relative redistribution of signaling toward myeloid and CD8^+^ T cell compartments; epithelial outgoing strength is reduced compared with NR.

NR samples. Sphere plot shows the Epithelia & Tumor_cell node with the highest total outgoing signaling strength; prominent epithelial→fibroblast, epithelial→myeloid, epithelial→endothelial and epithelial→neutrophil interactions are visible.


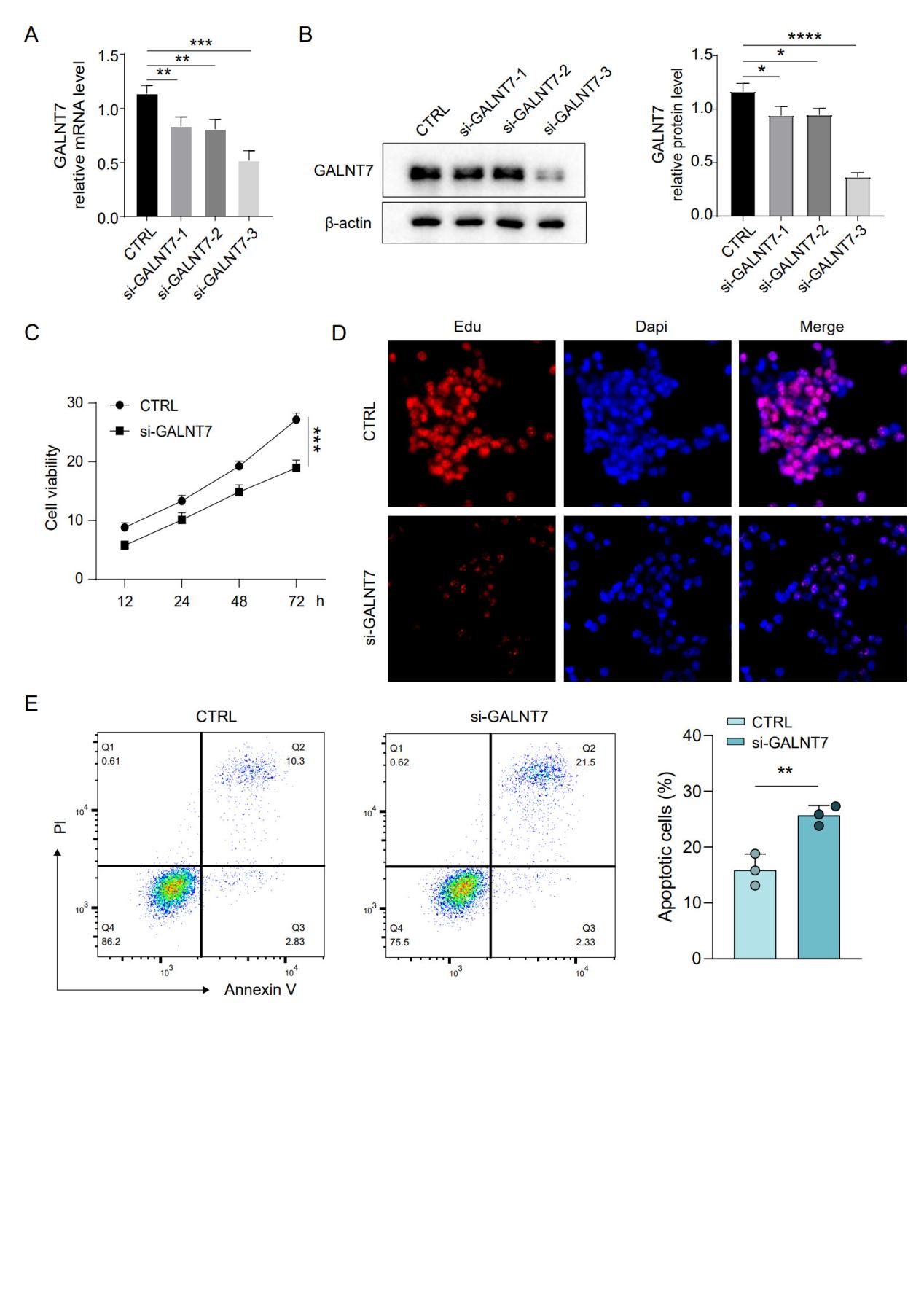


**Figure S7. GALNT7 knockdown suppresses Calu-3 cell proliferation and induces apoptosis**.

(A) qRT-PCR showing reduced GALNT7 mRNA expression after transfection with three independent siRNAs compared with control.

(B) with β-actin as the loading control; the right panel shows quantification of GALNT7 protein levels.

(C) Cell viability curve following si-GALNT7 transfection at indicated time points (12-72 h).

(D) Representative EdU incorporation images (EdU, DAPI, Merge) demonstrating impaired proliferation upon GALNT7 silencing.

(E) Annexin V/PI staining and flow-cytometry quantification showing significantly increased apoptotic fractions in GALNT7-depleted Calu-3 cells relative to control. Data represent mean±SEM from three independent experiments; *P < 0.05, **P < 0.01, ***P < 0.001 by Student’s t test.


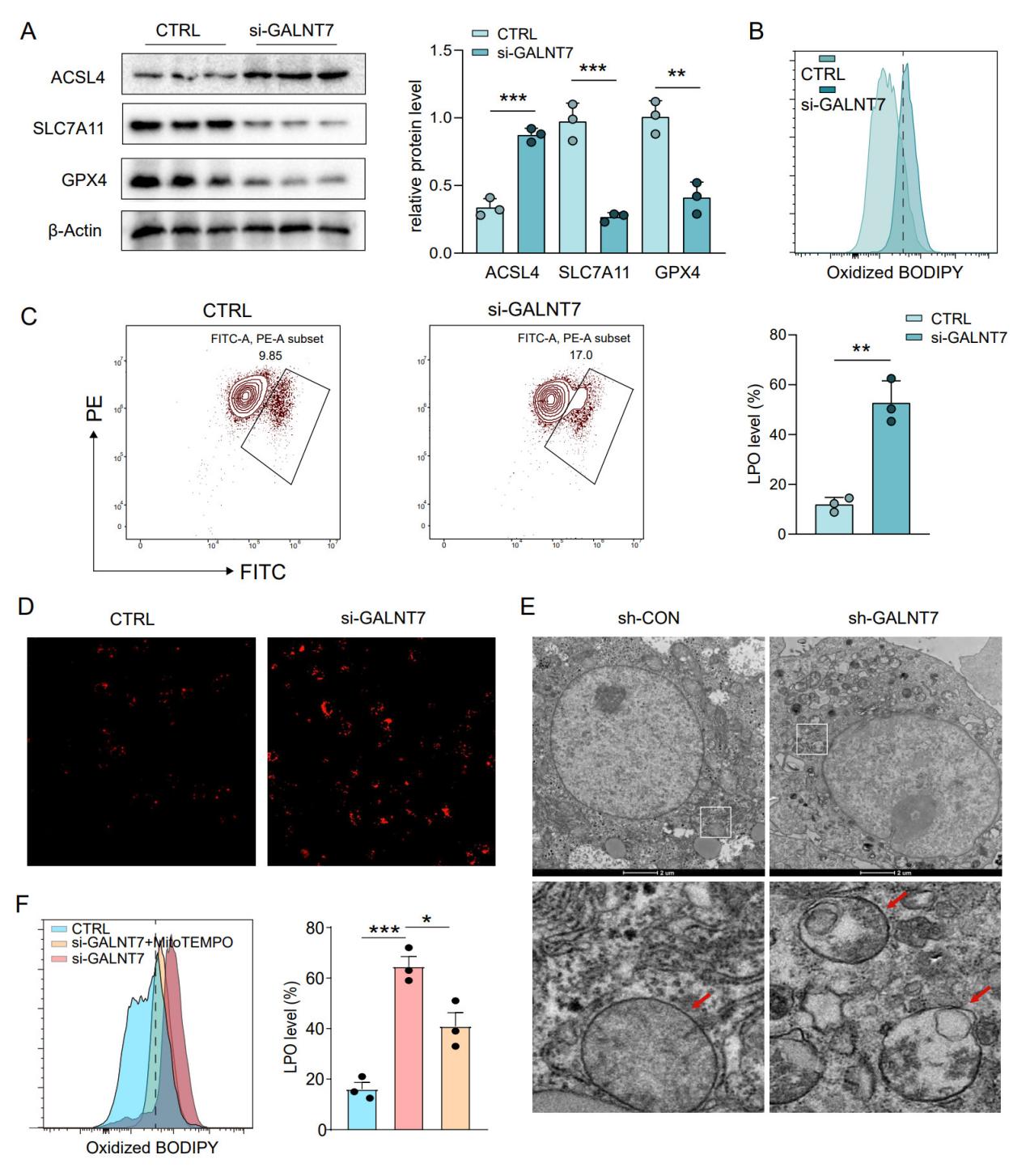


**Figure S8. GALNT7 knockdown activates ferroptosis signaling in Calu-3 cells.**

(A) Immunoblot analysis of ferroptosis-related proteins (ACSL4, SLC7A11, and GPX4) in control and si-GALNT7 Calu-3 cells; β-actin served as loading control. The right panel shows quantification of relative protein levels.

(B) Flow-cytometric measurement of oxidized BODIPY fluorescence indicating increased lipid-ROS accumulation upon GALNT7 silencing.

(C) Quantification of lipid-peroxidation (LPO) levels in control and si-GALNT7 cells.

(D) Fluorescence microscopy showing elevated oxidized BODIPY signal in si-GALNT7 Calu-3 cells compared with control.

(E) Transmission electron microscopy (TEM) analysis of Calu-3 cells after GALNT7 silencing. Enlarged panels show representative mitochondrial ultrastructural alterations. Arrowheads indicate mitochondria with shrinkage, increased membrane density, and disrupted or reduced cristae.

(F) Rescue experiment using mitochondrial antioxidant MitoTEMPO demonstrating reversal of LPO accumulation in GALNT7-depleted cells; the right panel shows corresponding quantification. Data represent mean±SEM from three independent experiments; *P < 0.05, **P < 0.01, ***P < 0.001 by Student’s t test.
