# Supplementary material for: Single‐Cell Reveal GALNT7‐Dependent Ferroptosis Suppression as a Mechanism of Immunotherapy Resistance in Non‐Small Cell Lung Cancer
Source: Adv Sci (Weinh). 2026 Jun 19:e76082. Online ahead of print. doi: 10.1002/advs.76082 (PMC13336806; doi:10.1002/advs.76082)
Supplement: Supplementary file 2 — Supporting File 2: advs76082‐sup‐0002‐FigureS1‐S8.zip. [file ADVS-9999-e76082-s001.zip › Figure S5.pdf]

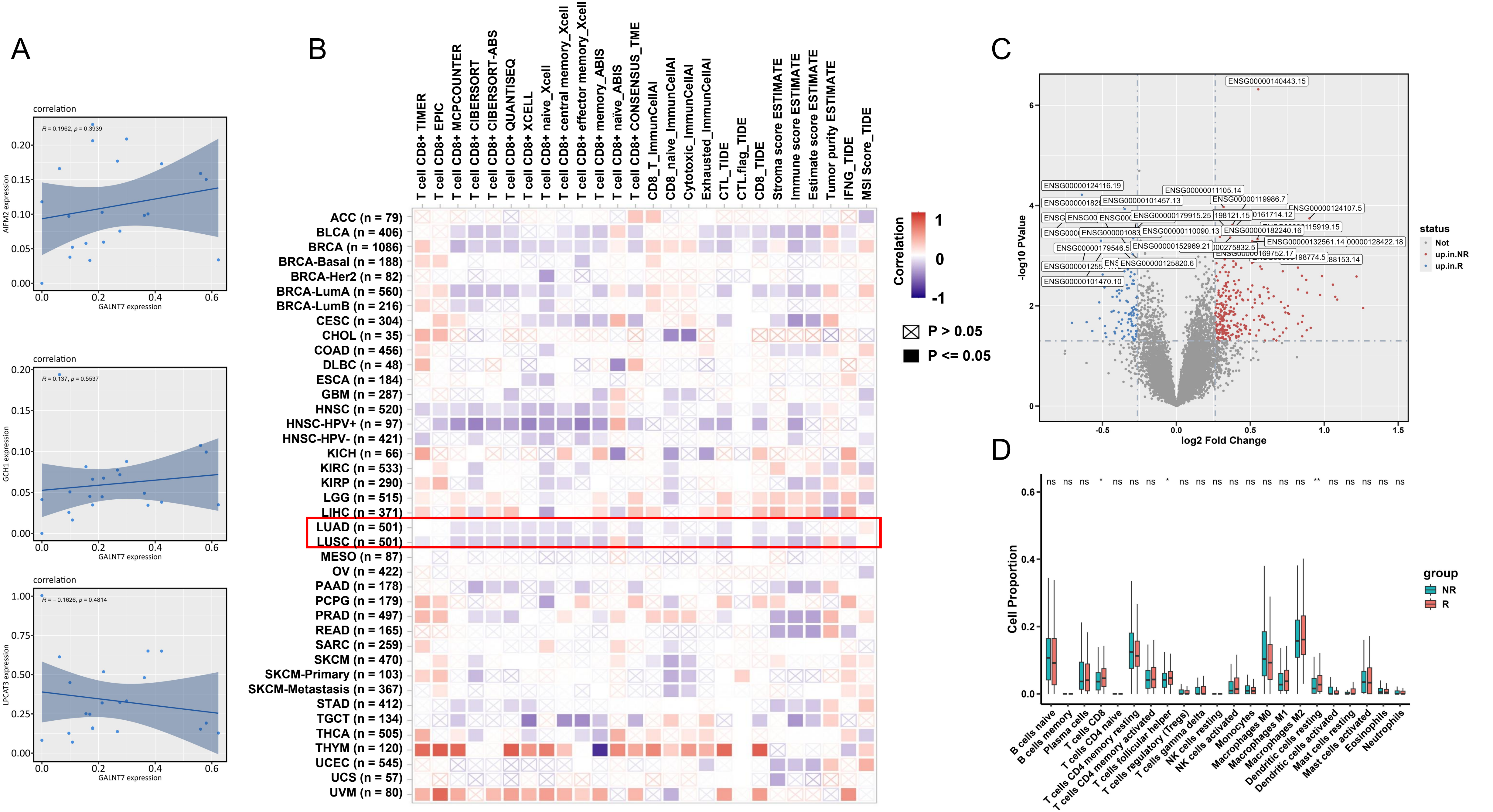

A
correlation
R = 0.1962, p = 0.3939
AIFM2 expression
GALNT7 expression
correlation
R = 0.137, p = 0.5537
GCH1 expression
GALNT7 expression
correlation
R = −0.1626, p = 0.4814
LPCAT3 expression
GALNT7 expression
B
T cell CD8+ TIMER
T cell CD8+ EPIC
T cell CD8+ MCPCOUNTER
T cell CD8+ CIBERSORT
T cell CD8+ CIBERSORT-ABS
T cell CD8+ QUANTISEQ
T cell CD8+ XCELL
T cell CD8+ naive_Xcell
T cell CD8+ central memory_Xcell
T cell CD8+ effector memory_Xcell
T cell CD8+ memory_ABIS
T cell CD8+ naïve_ABIS
T cell CD8+ CONSENSUS_TME
CD8_T_ImmunCellAI
CD8_naive_ImmunCellAI
Cytotoxic_ImmunCellAI
Exhausted_ImmunCellAI
CTL_TIDE
CTL.flag_TIDE
CD8_TIDE
Stroma score ESTIMATE
Immune score ESTIMATE
Estimate score ESTIMATE
Tumor purity ESTIMATE
IFNG_TIDE
MSI Score_TIDE
ACC (n = 79)
BLCA (n = 406)
BRCA (n = 1086)
BRCA-Basal (n = 188)
BRCA-Her2 (n = 82)
BRCA-LumA (n = 560)
BRCA-LumB (n = 216)
CESC (n = 304)
CHOL (n = 35)
COAD (n = 456)
DLBC (n = 48)
ESCA (n = 184)
GBM (n = 287)
HNSC (n = 520)
HNSC-HPV+ (n = 97)
HNSC-HPV- (n = 421)
KICH (n = 66)
KIRC (n = 533)
KIRP (n = 290)
LGG (n = 515)
LIHC (n = 371)
LUAD (n = 501)
LUSC (n = 501)
MESO (n = 87)
OV (n = 422)
PAAD (n = 178)
PCPG (n = 179)
PRAD (n = 497)
READ (n = 165)
SARC (n = 259)
SKCM (n = 470)
SKCM-Primary (n = 103)
SKCM-Metastasis (n = 367)
STAD (n = 412)
TGCT (n = 134)
THCA (n = 505)
THYM (n = 120)
UCEC (n = 545)
UCS (n = 57)
UVM (n = 80)
Correlation
1
0
-1
P > 0.05
P <= 0.05
C
ENSG00000140443.15
ENSG00000124116.19
ENSG00000011105.14
ENSG00000101457.13
ENSG00000119986.7
ENSG00000124107.5
ENSG00000179915.25
ENSG00000110090.13
ENSG00000182240.16
ENSG00000132561.14
ENSG00000179546.5
ENSG00000152969.21
ENSG00000125820.6
ENSG00000101470.10
-log10 PValue
log2 Fold Change
status
Not
up.in.NR
up.in.R
D
Cell Proportion
group
NR
R
B cells naive
B cells memory
Plasma cells
T cells CD8
T cells CD4 naive
T cells CD4 memory resting
T cells CD4 memory activated
T cells follicular helper
T cells regulatory (Tregs)
T cells gamma delta
NK cells resting
NK cells activated
Monocytes
Macrophages M0
Macrophages M1
Macrophages M2
Dendritic cells resting
Dendritic cells activated
Mast cells resting
Mast cells activated
Eosinophils
Neutrophils
