# Supplementary figures and images for: Single‐Cell Reveal GALNT7‐Dependent Ferroptosis Suppression as a Mechanism of Immunotherapy Resistance in Non‐Small Cell Lung Cancer
Source: Adv Sci (Weinh). 2026 Jun 19:e76082. Online ahead of print. doi: 10.1002/advs.76082 (PMC13336806; doi:10.1002/advs.76082)

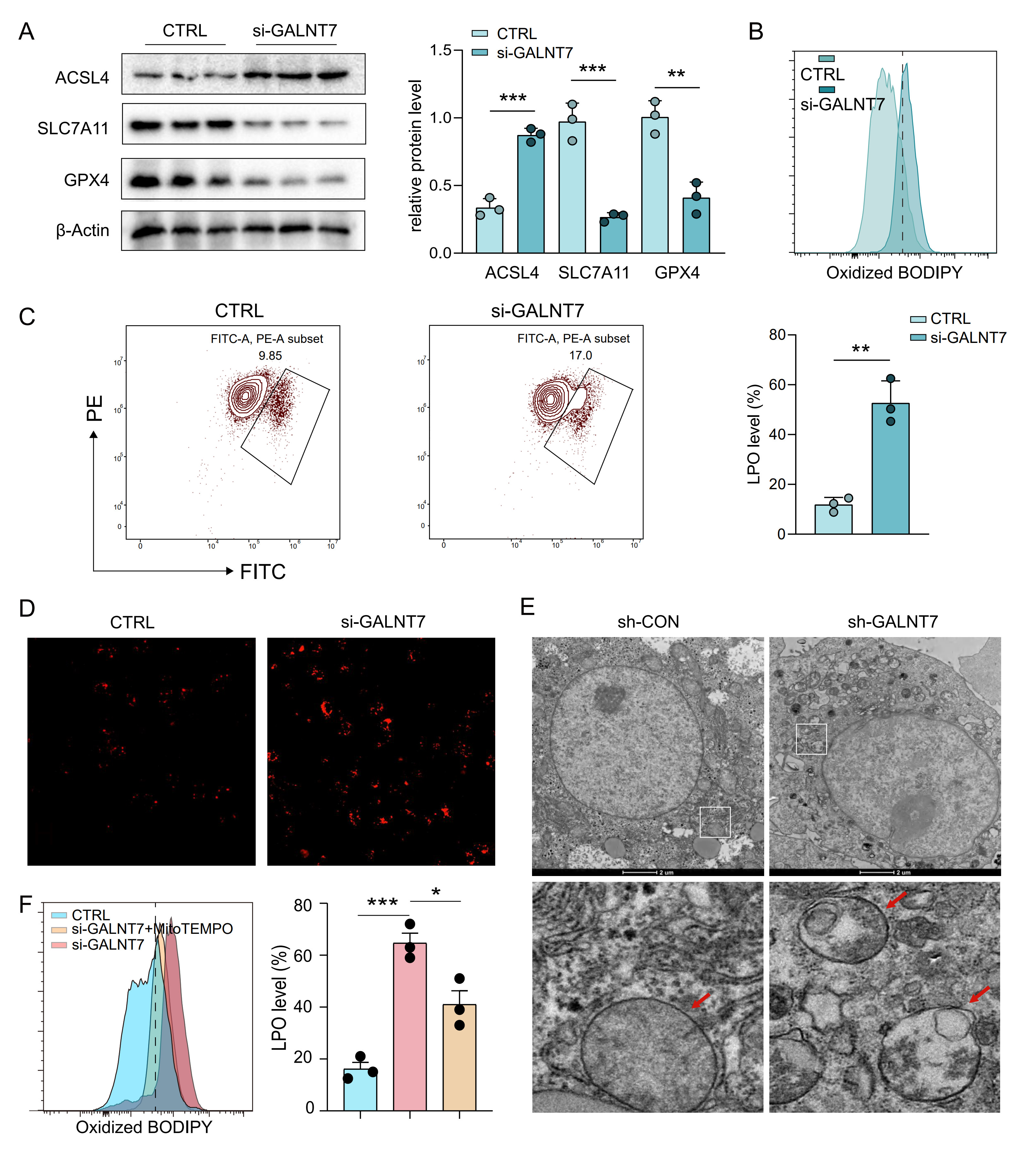

Supplement: Supplementary file 2 — Supporting File 2: advs76082‐sup‐0002‐FigureS1‐S8.zip. [file ADVS-9999-e76082-s001.zip › Figure S8.jpg]

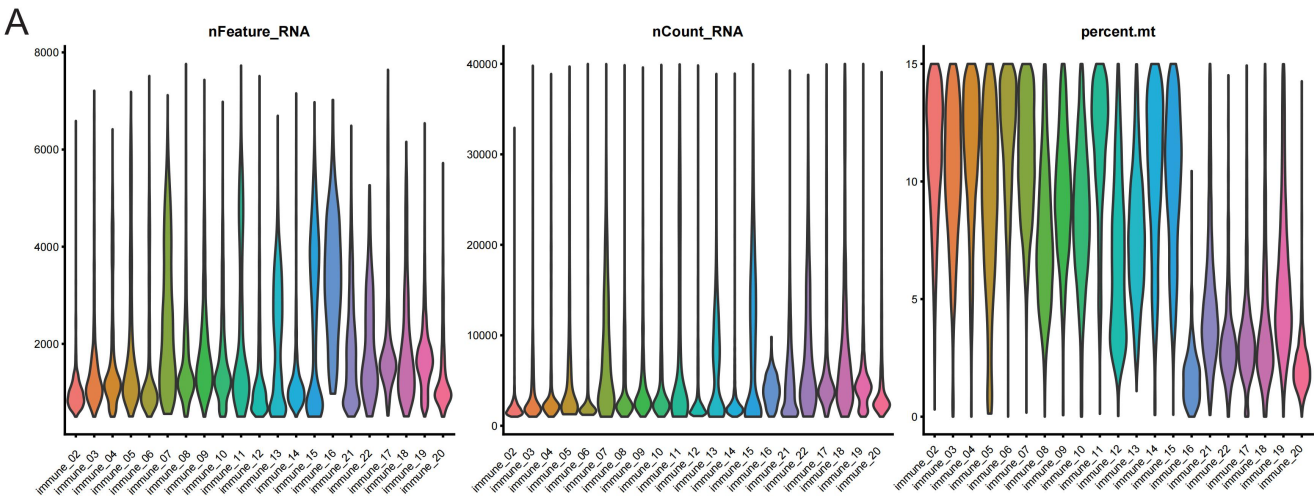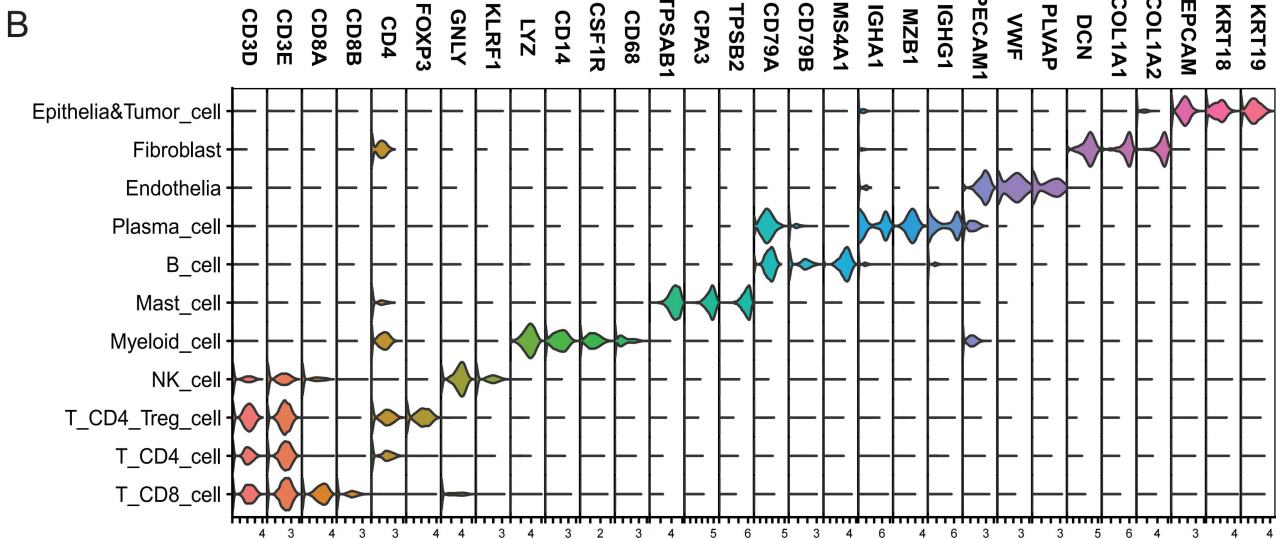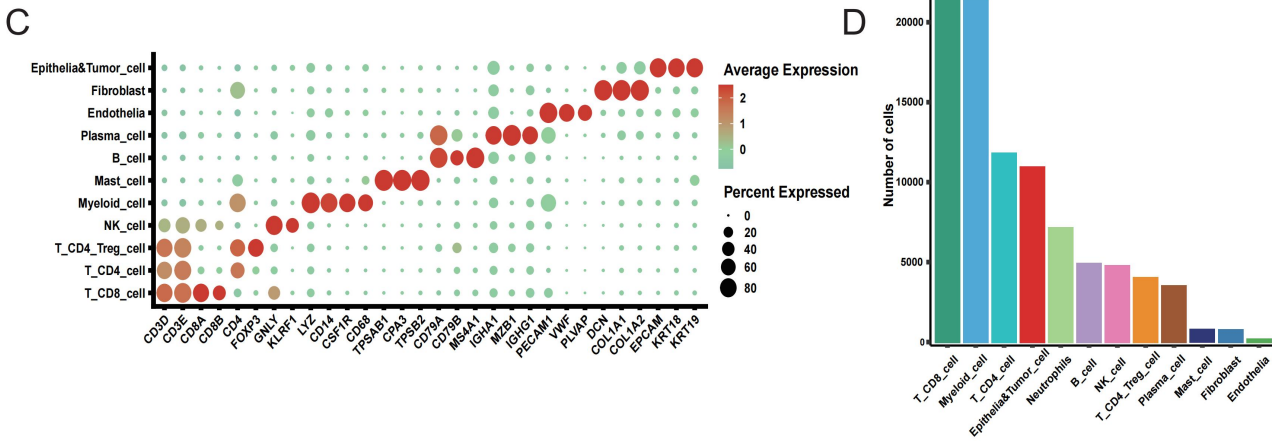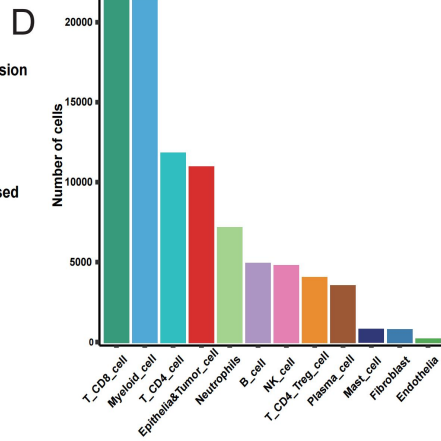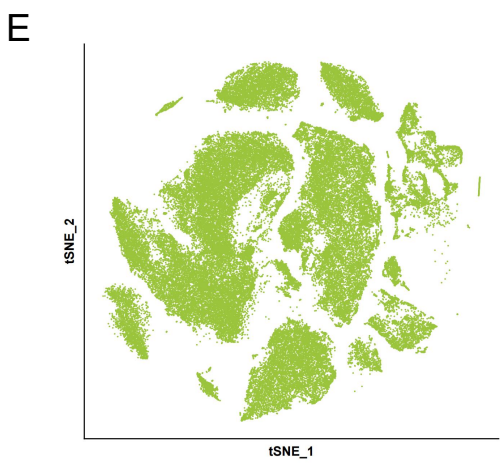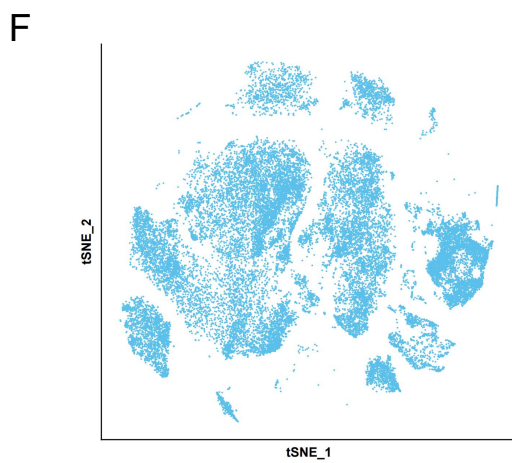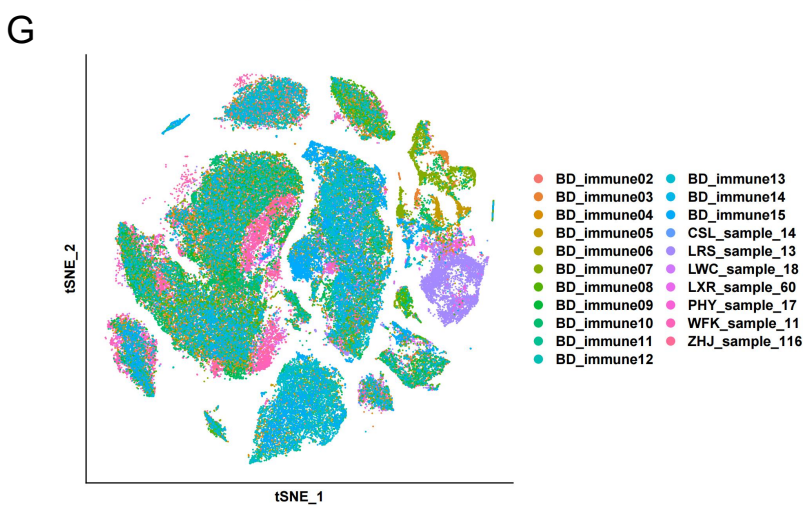

Supplement: Supplementary file 2 — Supporting File 2: advs76082‐sup‐0002‐FigureS1‐S8.zip. [file ADVS-9999-e76082-s001.zip › Figure S1.pdf]

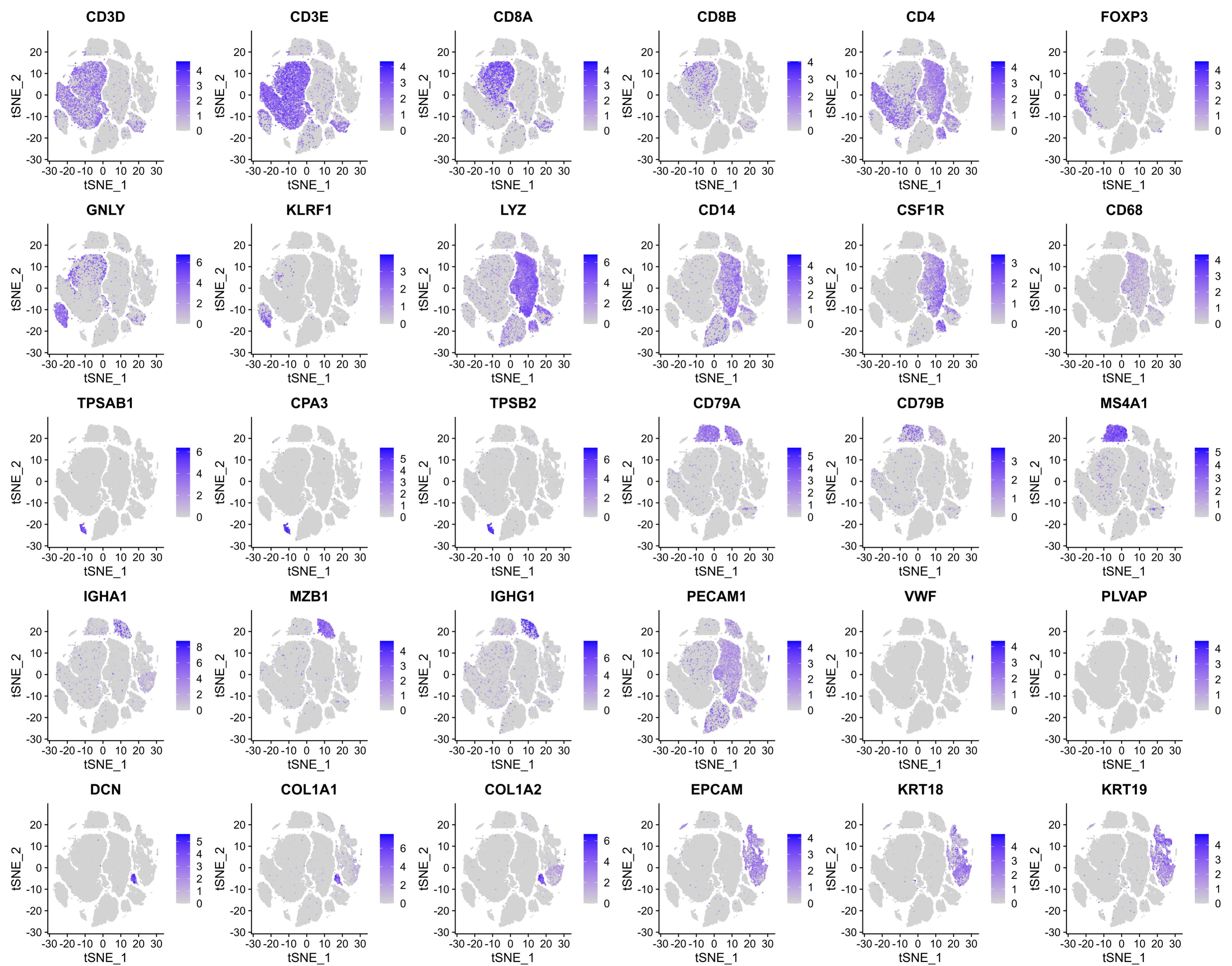

Supplement: Supplementary file 2 — Supporting File 2: advs76082‐sup‐0002‐FigureS1‐S8.zip. [file ADVS-9999-e76082-s001.zip › Figure S2.pdf]

inferCNV

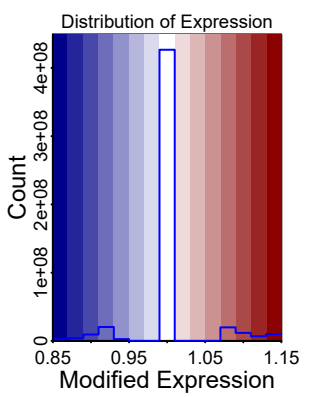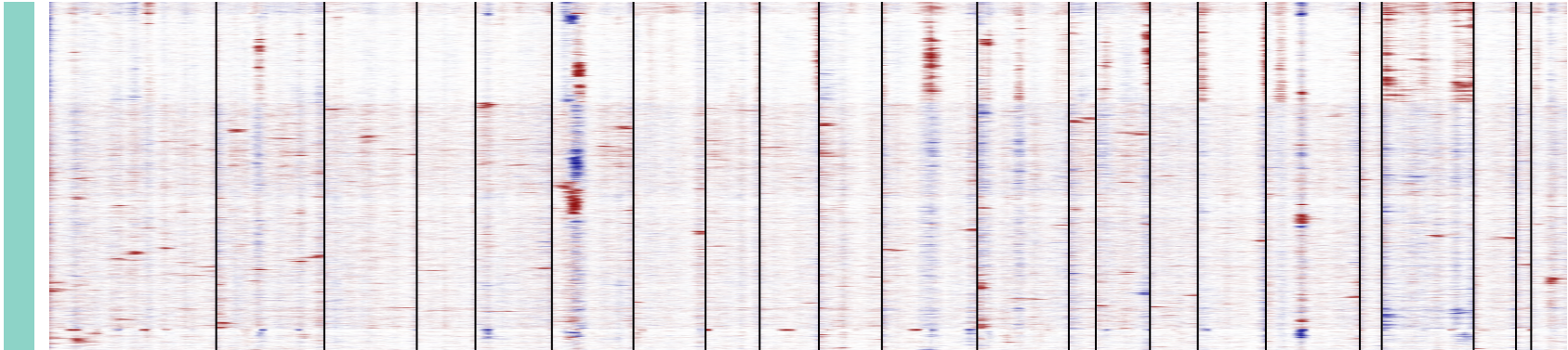

References (Cells)

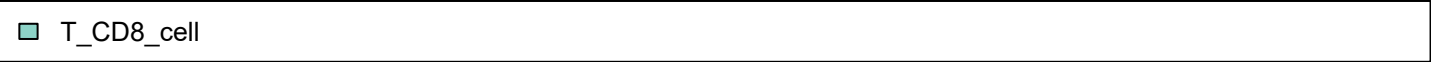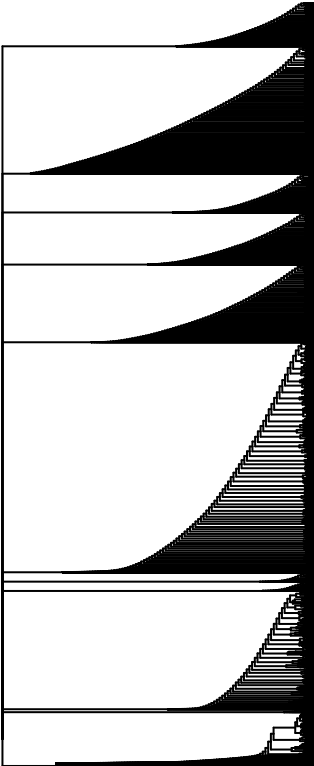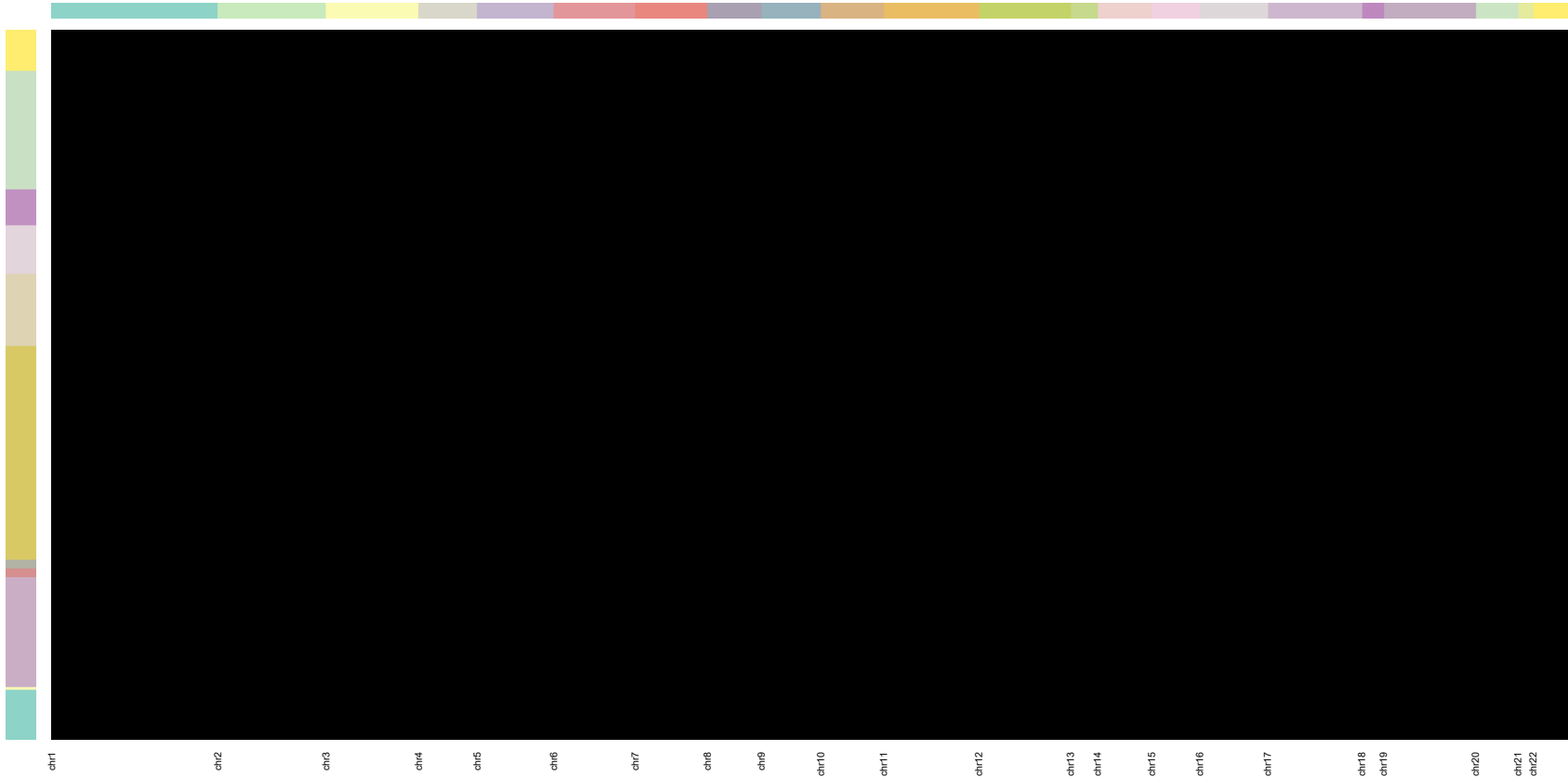

Observations (Cells)

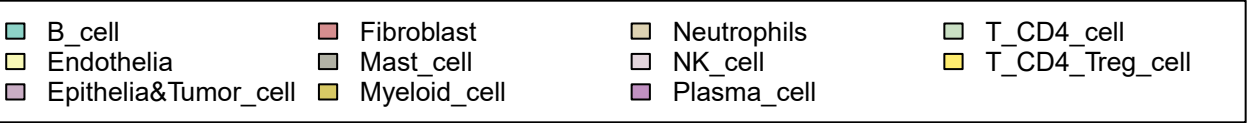

Supplement: Supplementary file 2 — Supporting File 2: advs76082‐sup‐0002‐FigureS1‐S8.zip. [file ADVS-9999-e76082-s001.zip › Figure S3.pdf]

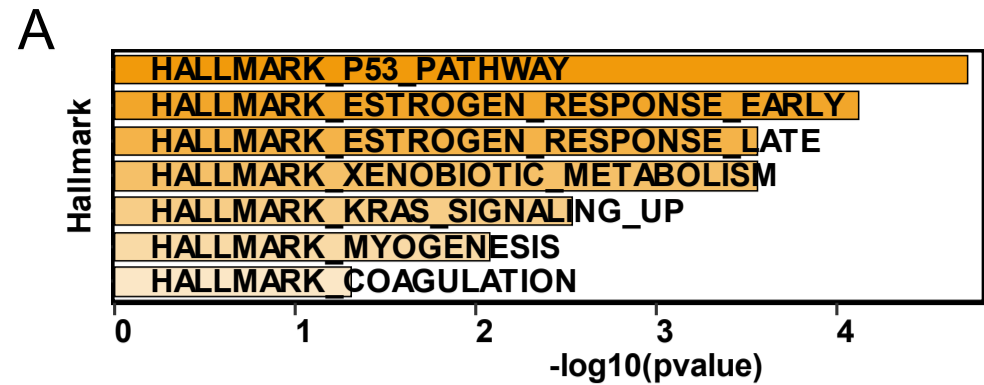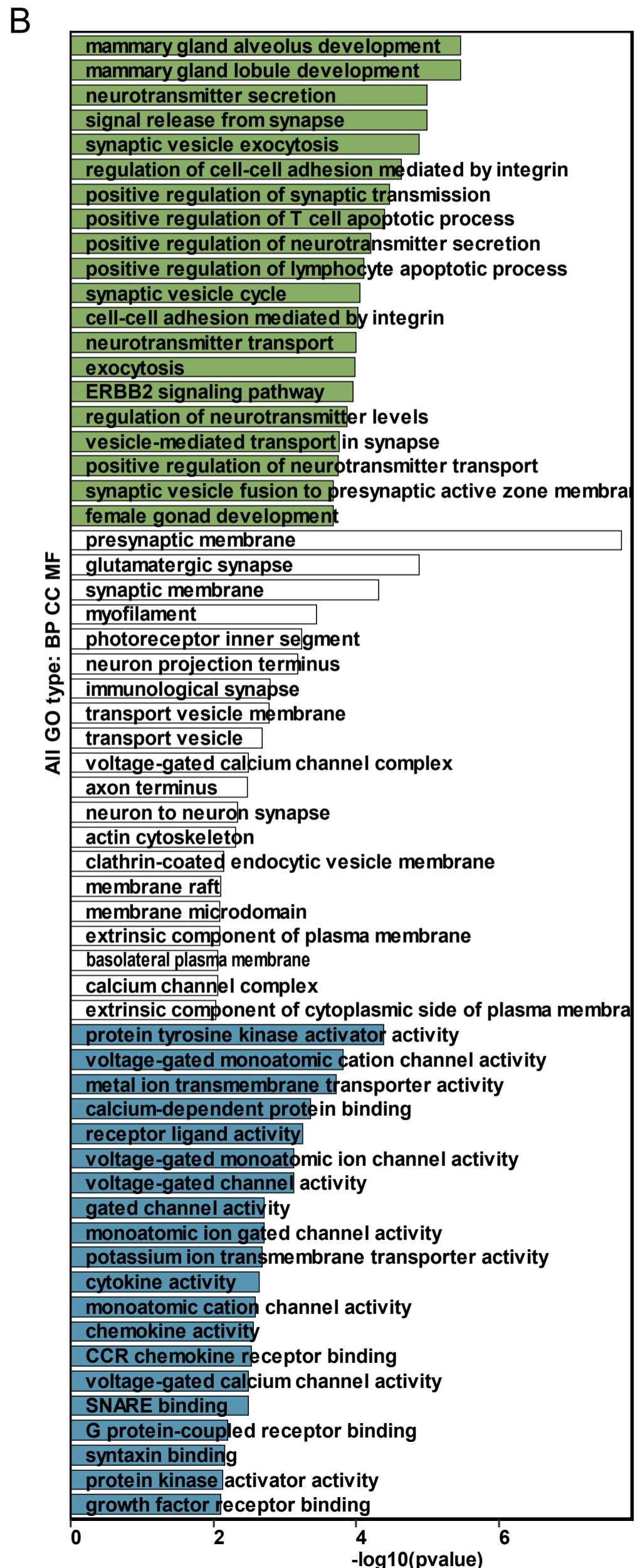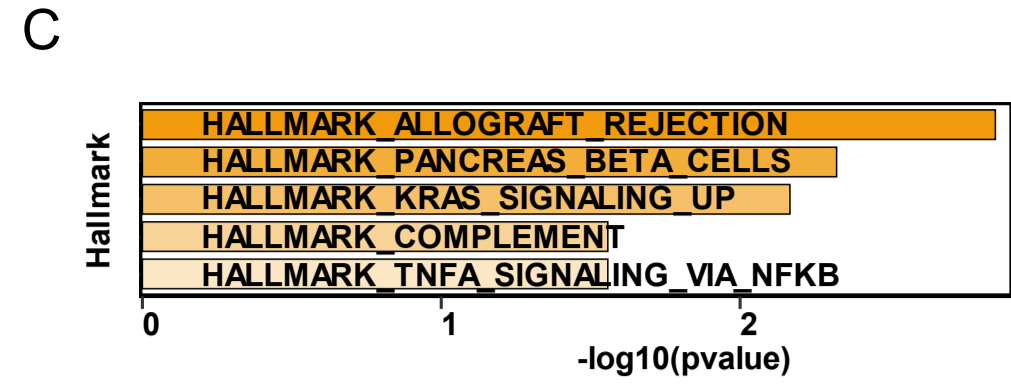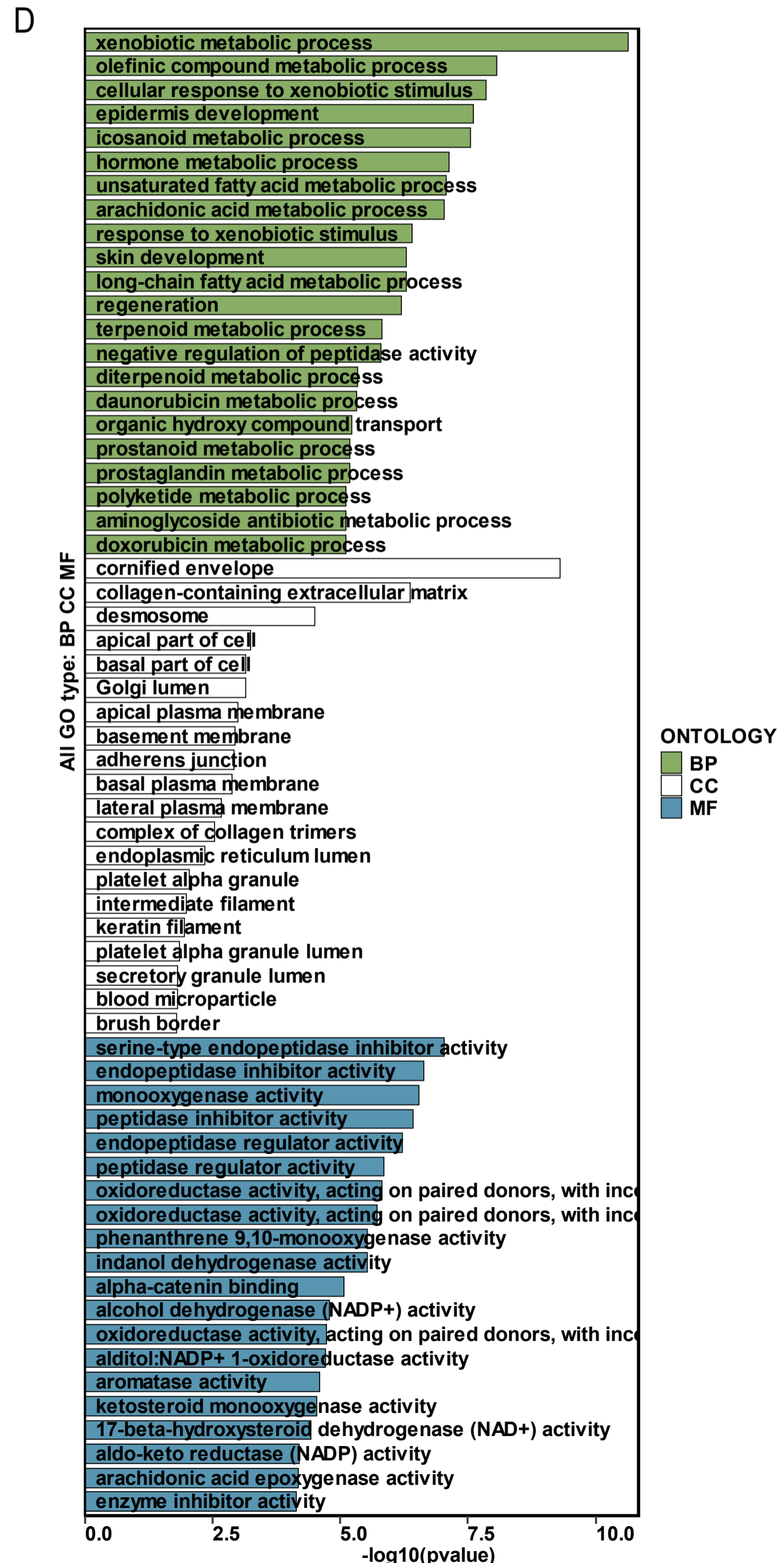

Supplement: Supplementary file 2 — Supporting File 2: advs76082‐sup‐0002‐FigureS1‐S8.zip. [file ADVS-9999-e76082-s001.zip › Figure S4.pdf]

A

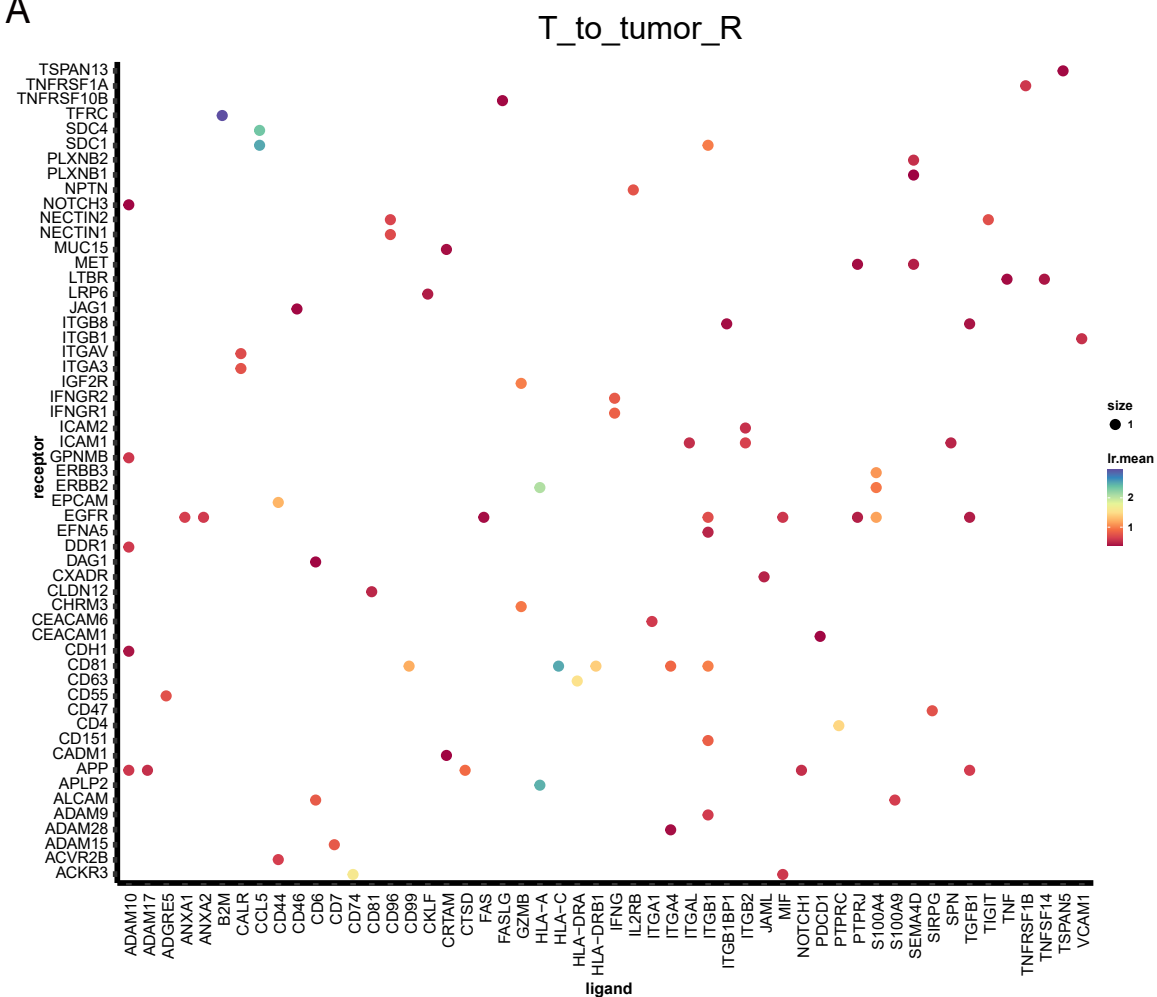

B

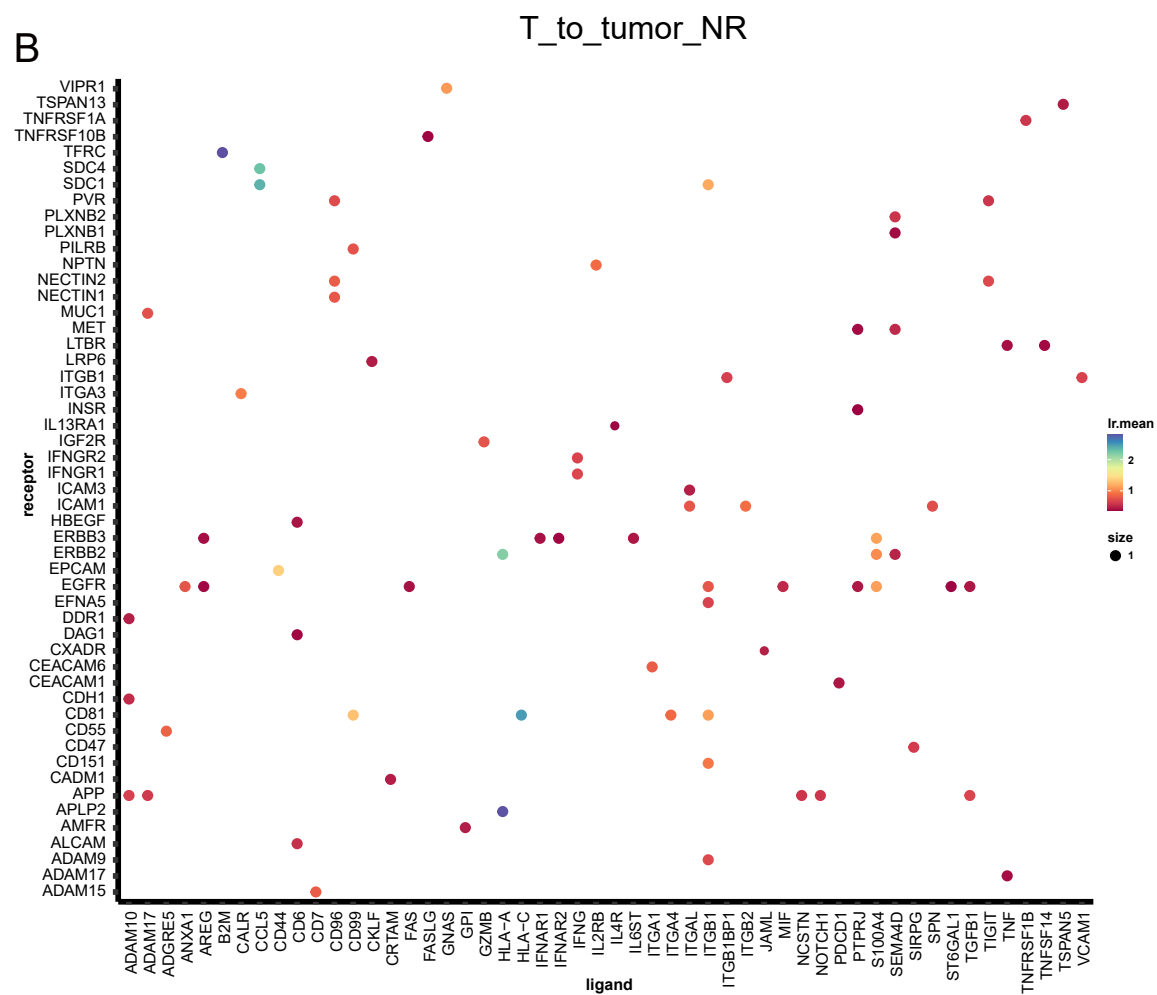

Supplement: Supplementary file 2 — Supporting File 2: advs76082‐sup‐0002‐FigureS1‐S8.zip. [file ADVS-9999-e76082-s001.zip › Figure S6.pdf]

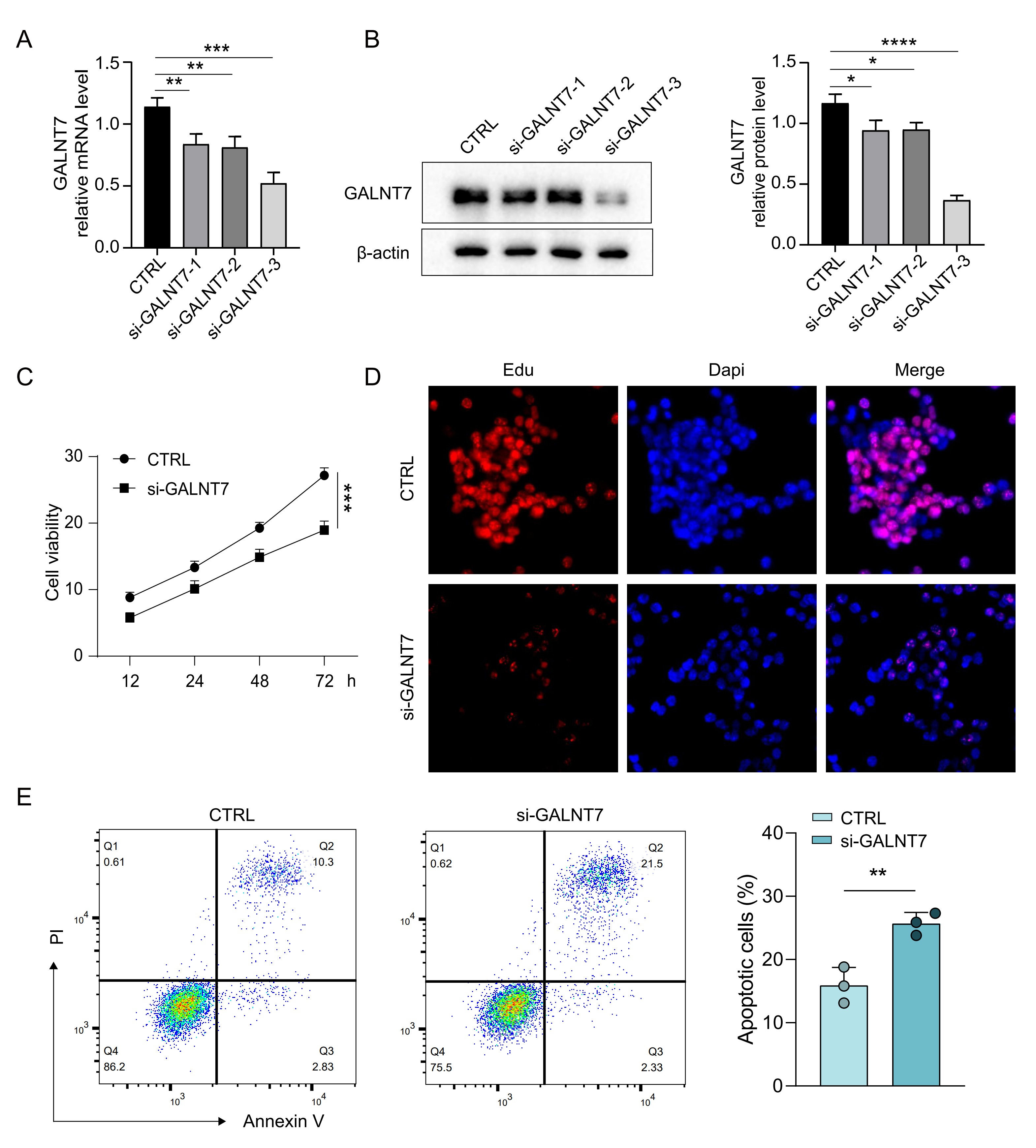

Supplement: Supplementary file 2 — Supporting File 2: advs76082‐sup‐0002‐FigureS1‐S8.zip. [file ADVS-9999-e76082-s001.zip › Figure S7.jpg]
